# Cell-type-specific interacting proteins collaborate to regulate the timing of Cyclin B protein expression in male meiotic prophase

**DOI:** 10.1242/dev.201709

**Published:** 2023-11-27

**Authors:** Catherine C. Baker, Lorenzo Gallicchio, Neuza R. Matias, Douglas F. Porter, Lucineh Parsanian, Emily Taing, Cheuk Tam, Margaret T. Fuller

**Affiliations:** ^1^ Department of Developmental Biology, Stanford University School of Medicine, Stanford, CA 94305, USA; ^2^ Program in Epithelial Biology, Stanford University School of Medicine, Stanford, CA 94305, USA

**Keywords:** *Drosophila*, Spermatogenesis, Meiosis, Translation, RNA, Cyclin B

## Abstract

During meiosis, germ cell and stage-specific components impose additional layers of regulation on the core cell cycle machinery to set up an extended G2 period termed meiotic prophase. In *Drosophila* males, meiotic prophase lasts 3.5 days, during which spermatocytes upregulate over 1800 genes and grow 25-fold. Previous work has shown that the cell cycle regulator Cyclin B (CycB) is subject to translational repression in immature spermatocytes, mediated by the RNA-binding protein Rbp4 and its partner Fest. Here, we show that the spermatocyte-specific protein Lut is required for translational repression of *cycB* in an 8-h window just before spermatocytes are fully mature. In males mutant for *rbp4* or *lut*, spermatocytes enter and exit meiotic division 6-8 h earlier than in wild type. In addition, spermatocyte-specific isoforms of Syncrip (Syp) are required for expression of CycB protein in mature spermatocytes and normal entry into the meiotic divisions. Lut and Syp interact with Fest independent of RNA. Thus, a set of spermatocyte-specific regulators choreograph the timing of expression of CycB protein during male meiotic prophase.

## Teaser

**Summary:** Timing of expression of conserved cell cycle regulator Cyclin B is controlled by multiple cell-type-specific proteins in *Drosophila* meiotic prophase male germ cells.

## INTRODUCTION

A key aspect of specialization during development and in stem cell lineages is regulation of the cell cycle: when to divide, whether to divide symmetrically or asymmetrically, whether to exit the cell cycle completely or to undertake unusual cell cycles. Unconventional cell cycles include those that skip M phase, leading to polyploidy, and meiosis, which features an extended G2 cell cycle phase termed meiotic prophase. Both extrinsic cues and intrinsic, lineage-specific factors feed into regulation of the core cell cycle machinery to generate cell-type and tissue-specific control of the cell cycle. For example, the well-studied mechanisms that regulate the length of meiotic prophase in females are strongly influenced by extrinsic factors such as hormonal control. In human females, meiotic prophase can last 12-50 years. However, less is known about the mechanisms that regulate meiotic prophase in males, where it tends to last a specified time depending on the species: 12 days in mouse and 3.5 days in *Drosophila*.

**Fig. 1.**
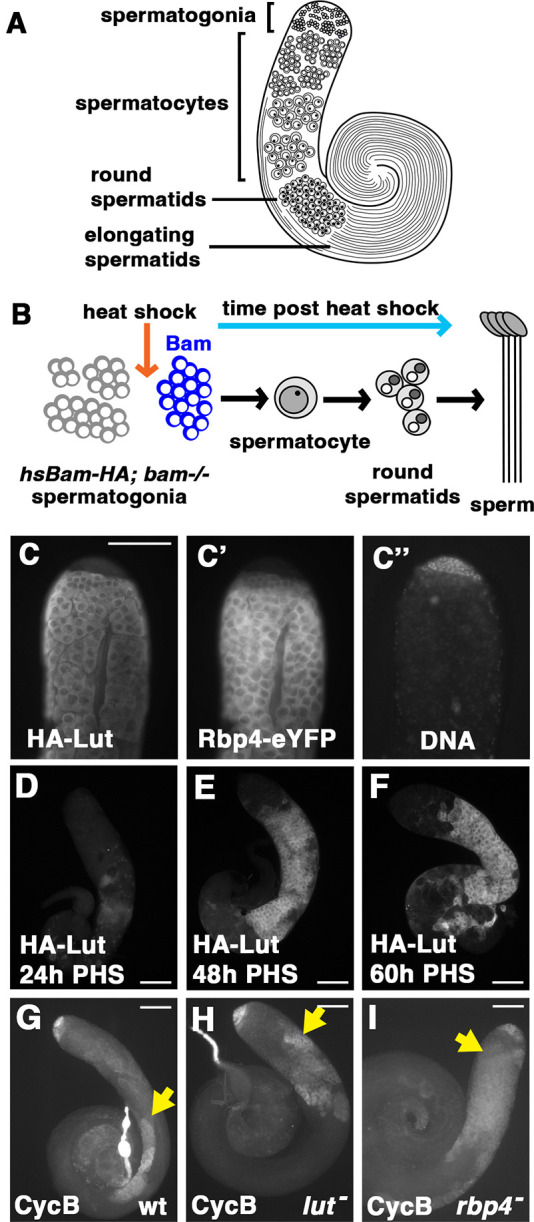
**Lutin is expressed early in spermatocyte development and is required for repression of *cycB* translation in still-immature spermatocytes.** (A) Illustration of testis with major germline cell types labeled. (B) Schematic of the hs-Bam differentiation time-course. (C-C″) Immunofluorescence image of the apical tip of a testis from a male carrying HA-Lut and Rbp4-eYFP transgenes, stained with anti-HA (C), anti-GFP (C′) and DAPI (C″). (D-F) Anti-HA staining of apical tips of testes from flies expressing HA-Lut in the hs-Bam time-course collected at 24 h post-heat-shock (PHS) (D), 48 h PHS (E) and 60 h PHS (F). (G-I) Anti-CycB staining of wild-type (wt) (G), *lut* (H) and *rbp4* (I) mutant testes. Yellow arrows indicate onset of CycB protein expression in spermatocytes. Scale bars: 100 µm.

Studies of the *Drosophila* male germline are beginning to reveal how intrinsic factors controlled by the male germ cell developmental program impose layers of regulation on core cell cycle machinery components during the specialized cell cycle of meiosis. In *Drosophila* testes, germ cells are arrayed in a roughly spatiotemporal order originating from germline stem cells at the apical tip of the testis (Fig. 1A). When germline stem cells divide they leave a stem cell daughter at the hub and release a daughter cell (gonialblast) that will give rise to all of the later germ cell types. The gonialblast initiates four rounds of synchronous mitotic divisions with incomplete cytokinesis, producing 16 interconnected germ cells that undergo pre-meiotic S phase. During the subsequent meiotic prophase, the resulting spermatocytes grow 25-fold in volume and turn on the massive transcription program required for post-meiotic spermatid differentiation. The 3.5-day meiotic G2 phase is characterized by developmental mechanisms that delay the appearance of core cell cycle regulatory proteins such as Cyclin B (CycB) and the Cdc25 phosphatase Twine (Twe) until just before they are needed for the meiotic divisions (White-Cooper et al., 1998; Maines and Wasserman, 1999).

Here, we show that several cell-type-specific interacting proteins and protein isoforms expressed in spermatocytes regulate the timing of CycB protein expression during male meiotic prophase in *Drosophila*. We had previously shown that the RNA-binding protein Rbp4 and its binding partner Fest were required to repress translation of *cycB* in immature spermatocytes. Rbp4 bound the 130 nt *cycB* 3′ untranslated region (UTR) expressed in spermatocytes in biotin pulldown assays, suggesting that Rbp4 might act directly on *cycB* RNA to block its translation (Baker et al., 2015). We now identify a novel protein Lutin (Lut; encoded by *CG1690*, hereafter known as *lut*), co-expressed at the onset of spermatocyte differentiation with Rbp4 and Fest, that interacts with Fest (and with Rbp4, via Fest) independently of RNA. We show that Lut is also required for cell-type-specific translational repression of *cycB* in spermatocytes, although at a later stage. CycB protein appears about 50 h early in spermatocytes of males mutant for *rbp4*, but only about 8 h early in males mutant for *lut*. Spermatocytes mutant for either *rbp4* or *lut* enter and exit the meiotic divisions about 6-8 h earlier than in wild type, indicating that within the narrow window of late-stage spermatocytes, premature expression of CycB protein may help promote early meiotic entry. We also show that testis-specific isoforms of Syp, the homolog of mammalian SYNCRIP, are required for expression of CycB protein in mature spermatocytes and for normal meiotic progression and post-meiotic differentiation. A cytoplasmic isoform of Syp expressed in spermatocytes binds the *cycB* 5′ and 3′ UTRs and interacts with Rbp4, Fest and Lut via Fest throughout spermatocyte development. Data from the *syp* mutant suggest a role for Syp in stabilizing the *cycB* RNA, and results from *rbp4 syp* and *lut syp* double mutants point to possible models for how the three proteins might act in series or in parallel to regulate *cycB* RNA stability and translation during the extended meiotic G2 prophase.

## RESULTS

### Lutin associates with Rbp4 and Fest

To identify binding partners of Rbp4 and Fest that could help regulate stage-specific *cycB* translation, we immunoprecipitated either Rbp4-eYFP or eYFP-Fest from testis extracts and analyzed the results of immunoprecipitation-mass spectrometry (IP-MS). IP-MS hits were ranked by the ratio of the total number of peptides recovered to the size of the protein predicted by FlyBase (Table 1). As the immunoprecipitations were not performed in the presence of RNAse A, proteins without RNA-binding domains were of particular interest as most likely to co-immunoprecipitate due to protein-protein interactions instead of binding to a common RNA. Lutin co-immunoprecipitated with both Rbp4-eYFP and eYFP-Fest (Table 1) and had no known RNA-binding domains.

**Table 1.**
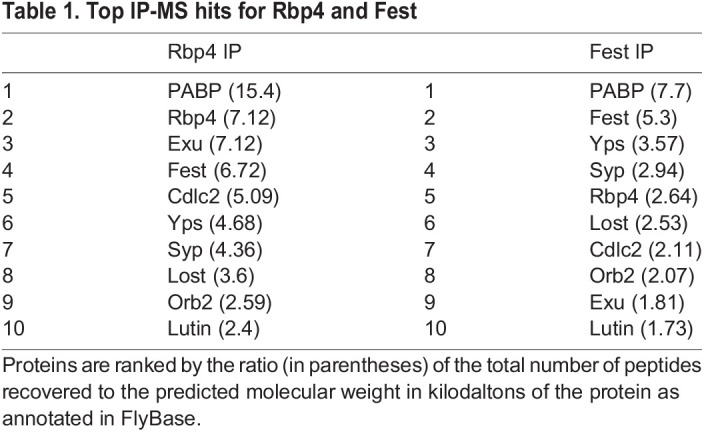
Top IP-MS hits for Rbp4 and Fest

### Lutin represses premature translation of *cycB* in spermatocytes

The *Drosophila* gene *lut* encodes a 15 kD predicted protein, with no currently recognized domains, that is conserved in most Drosophilid species, notably the entire subgenus *Sophophora* as well as Hawaiian *Drosophila*. The only homolog found outside this genus was in *Scaptodrosophila lebanonensis*. We named the gene *lutin* after the small and pleasantly-mischievous French house-elf. Expression of Lutin initiates early in spermatocyte development, as first revealed by RNA-seq data from a heat-shock Bam (hs-Bam) differentiation time-course. This technique exploits the fact that *bam* mutant testes accumulate spermatogonia, but following a short pulse of wild-type Bam expression under the control of a heat-shock promoter, germ cells march through differentiation in rough synchrony (Kim et al., 2017; illustrated in Fig. 1B). RNA-seq analysis of testes from *hsBam; bam* flies subjected to a single pulse of heat shock showed that *lut* RNA was upregulated over 1000-fold by 48 h post-heat-shock (PHS) compared with testes from *bam* flies heat-shocked and cultured in parallel (Kim et al., 2017). A transgene composed of the endogenous *lut* promoter driving a *lut* cDNA with a 3×HA N-terminal tag rescued male sterility of *lut* mutants (Materials and Methods) and showed expression of HA-Lut protein starting in very early spermatocytes (Fig. 1C), in a pattern similar to Rbp4-eYFP (Fig. 1C′); notably, expression of the two reporters did not overlap with the bright DNA signal seen in spermatogonia (Fig. 1C”). Like Rbp4-eYFP, the HA-Lut protein was cytoplasmic. Consistent with the time-course RNA-seq data, expression of HA-Lut from the transgene was not detected in germ cells at 24 h PHS but was robustly expressed in differentiating spermatocytes at 48 h and 60 h PHS (Fig. 1D-F).

Function of Lutin is required to repress premature expression of CycB protein in spermatocytes. A loss-of-function allele of *lut* (*lut^1^*) made by CRISPR microdeletion encodes a Lut protein missing nearly two thirds of its sequence. Immunofluorescence staining revealed expression of CycB protein in spermatocytes closer to the apical tip of the testis in *lut^1^/Df* males (hereafter referred to as *lut* mutants) than in wild type (Fig. 1G,H), although not as apical (early) as seen in testes from males mutant for *rbp4* (Fig. 1I).

### Lut interacts with Rbp4 via binding to Fest

**Fig. 2.**
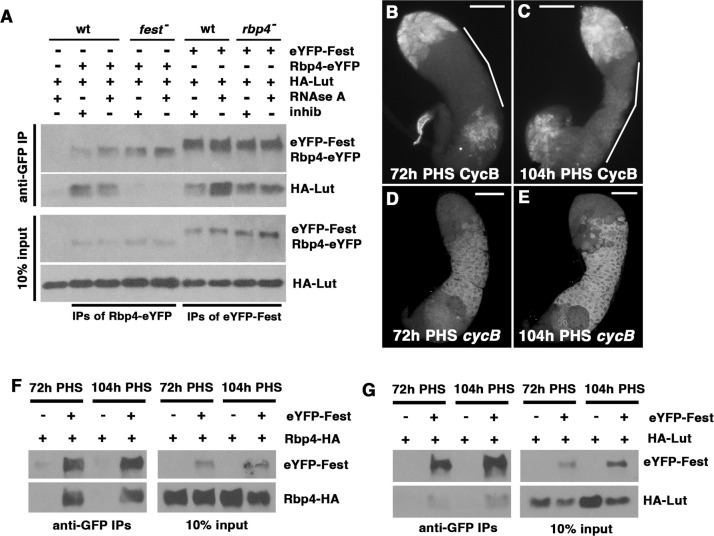
**Lutin interacts with Fest and with Rbp4 dependent on Fest.** (A) Western blots probed with anti-GFP or anti-HA showing proteins immunoprecipitated with anti-GFP from testis extracts from flies expressing HA-Lut and either Rbp4-eYFP (lanes 2-5) or eYFP-Fest (lanes 6-9). Negative control: HA-Lut alone (lane 1). RNAse A or RNAse inhibitor was added as indicated. Flies used in lanes 4-5 were *fest* mutants. Flies used in lanes 8-9 were *rbp4* mutants. (B,C) Testes from the hs-Bam time-course immunostained with anti-CycB. (B) 72 h post-heat-shock (PHS). Segmented line: region of the testis containing spermatocytes. (C) 104 h PHS. Segmented line: CycB-positive spermatocytes. (D,E) smFISH with *cycB* probes on 72 h PHS (D) and 104 h PHS testes (E). (F) Western blots probed with anti-GFP or anti-HA of proteins immunoprecipitated with anti-GFP from testis extracts of flies expressing eYFP-Fest and Rbp4-HA. Samples were dissected at 72 h or 104 h PHS as indicated, and all samples had RNAse A added. Negative control: Rbp4-HA alone. (G) Western blots probed with anti-GFP or anti-HA of proteins immunoprecipitated with anti-GFP from testis extracts of flies expressing eYFP-Fest and HA-Lut. Samples were dissected at 72 h and 104 h PHS, and all samples had RNAse A added. Negative control: HA-Lut alone. Scale bars: 100 µm.

The Lut protein physically interacts with Fest, independently of RNA. Immunoprecipitation of eYFP-Fest with anti-GFP from testis extracts brought down HA-Lut, both in the presence or absence of RNAse A (Fig. 2A, right side). Immunoprecipitation of HA-Lut with eYFP-Fest held true, even in testes mutant for the Fest-interacting protein Rbp4. In contrast, although immunoprecipitation of Rbp4-eYFP brought down HA-Lut from wild-type extracts in the presence and absence of RNAse (Fig. 2A, left side), HA-Lut did not co-immunoprecipitate with Rbp4 in a *fest* mutant background, indicating that Rbp4 requires Fest for its interaction with Lut. Together these data suggest that Rbp4, Fest, and Lut associate with each other with Fest at the center.

To test whether the switch from *cycB* translational repression to production of CycB protein might be accompanied by a substantial change in the interactions of Lut, Fest and Rbp4, we repeated the co-immunoprecipitation experiments in the hs-Bam time-course genetic background. In the hs-Bam time-course, CycB protein was not detected by immunostaining in spermatocytes at 72 h PHS (Fig. 2B, white line) but was detected in spermatocytes at 104 h PHS (Fig. 2C, white line), just before entry into the first meiotic division, whereas the *cycB* RNA was detected at both timepoints by single molecule fluorescence *in situ* hybridization (smFISH) (Fig. 2D,E). Immunoprecipitation of eYFP-Fest brought down Rbp4-HA and HA-Lut at both 72 h and 104 h PHS (Fig. 2F,G), indicating that the appearance of CycB protein in mature spermatocytes is not likely to be due to dissociation of either Rbp4 or Lut from Fest.

### Lut is required for translational repression of *cycB* in an 8-h window just before spermatocytes are fully mature

**Fig. 3.**
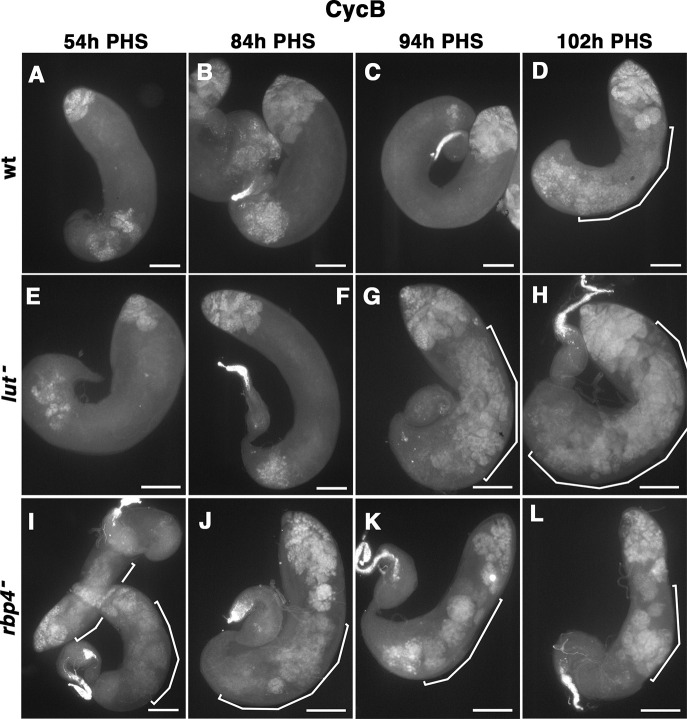
**Ectopic expression of CycB occurs later in *lut* mutants than it does in *rbp4* mutants.** (A-L) Testes from the hs-Bam time-course stained with anti-CycB. (A-D) wild type (E-H) *lut* (I-L) *rbp4*. Segmented lines: CycB-positive spermatocytes. PHS, post-heat-shock. Scale bars: 100 µm.

Analysis of CycB protein expression in the context of the hs-Bam time-course confirmed that loss of function of Rbp4 and Lut had different effects on the timing of CycB protein expression. Testes from either wild type, *rbp4* or *lut* mutants crossed into the hs-Bam time-course background were collected at 54 h, 84 h, 94 h and 102 h PHS and immunostained for CycB. In wild-type time-course testes, CycB protein was not detected in spermatocytes at the first three timepoints but was detected in spermatocytes at 102 h (Fig. 3A-D). In *lut* mutant time-course testes, CycB protein was detected at 94 h and 102 h but not at 54 h or 84 h PHS (Fig. 3E-H). Notably, the time at which Lut function was required to repress *cycB* translation was much later than the appearance of the Lut protein (48 h PHS, Fig. 1D). In *rbp4* time-course testes, in contrast, expression of CycB protein was detected at all four time points (Fig. 3I-L). Levels of *cycB* RNA detected were comparable across the three genotypes (Fig. S1).

### Spermatocytes in *lut* or *rbp4* mutants advance to and exit the meiotic divisions 6-8 h earlier than in wild type

**Fig. 4.**
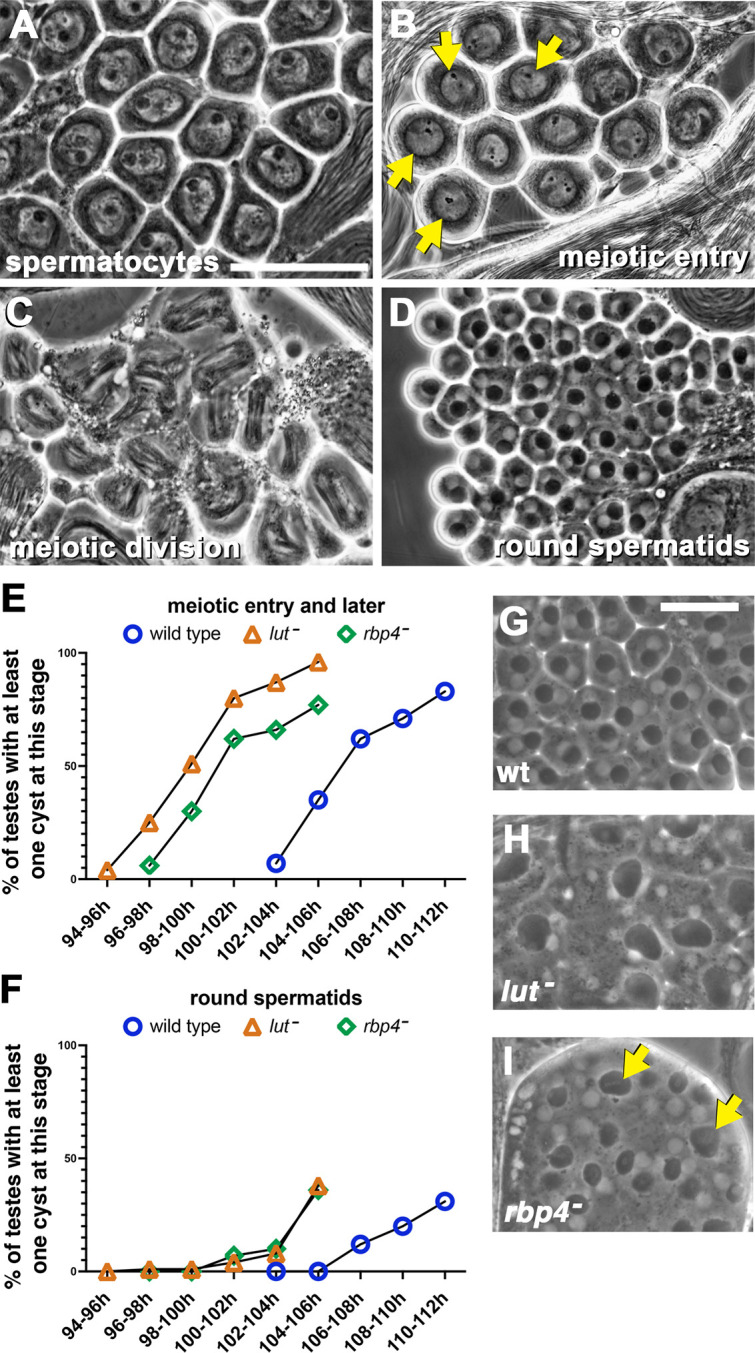
***lut* and *rbp4* mutant spermatocytes enter and exit the meiotic divisions earlier than in wild type.** (A-D) Phase-contrast imaging of unfixed wild-type (wt) testis squashes. (A) Mature spermatocytes. (B) Spermatocytes initiating meiotic entry, showing rounded nuclei (arrows) and crumbly nucleoli. (C) Cells in the first meiotic division. (D) Round spermatids, each with a small phase-light nucleus and phase-dark mitochondrial derivative. (E,F) Graphs showing percentage of testes from wt, *lut* mutants and *rbp4* mutants with at least one cyst at meiotic entry or later (i.e. including stages shown in B-D) (E) or at least one cyst with round spermatids (F). *N*=100 for each timepoint/genotype combination. (G-I) Phase-contrast imaging of testis squashes with round spermatids from wild type (G), *lut* mutants (H) and *rbp4* mutants (I). Arrows in I show larger-than-normal nebenkern, indicating at least one failed meiotic cytokinesis. Scale bars: 50 µm (A-D); 25 µm (G-I).

Germ cells in *lut* or *rbp4* mutant males entered and exited the meiotic divisions earlier than their wild-type counterparts. In live squashed preparations viewed by phase-contrast microscopy, wild-type mature spermatocytes had a boxy nucleus and a phase-dark, round nucleolus (Fig. 4A), whereas spermatocytes just entering the first meiotic division had a rounder nucleus and a shrunken, crumbly nucleolus (Fig. 4B). Dividing cells were recognizable by their phase-dark array of mitochondria associated with the meiotic spindle (Fig. 4C), and post-meiotic early round spermatids had a characteristic phase-light round nucleus paired with a phase-dark round mitochondrial derivative (Fig. 4D). Testes from wild-type, *lut* or *rbp4* mutant males in the hs-Bam time-course background were scored for presence of germline cysts containing cells that had advanced at least as far as meiotic entry (Fig. 4E) and for presence of cysts of round spermatids (Fig. 4F). At 106-108 h PHS, more than half of wild-type time-course testes contained at least one cyst showing meiotic entry, whereas *lut* and *rbp4* mutants hit that benchmark at 98-100 h PHS and 100-102 h PHS, respectively. Appearance of cysts of round spermatids ran about 6 h earlier in both *lut* and *rbp4* mutants compared with wild-type testes, as about one-third of testes from those two mutant genotypes contained at least one cyst with round spermatids at 104-106 h PHS (versus 110-112 h PHS in wild type).

Loss of function of *lut* resulted in defects in cytokinesis after meiosis I and meiosis II. In wild type, the meiotic divisions produced round spermatids with one nucleus and one phase-dark mitochondrial derivative, with these two structures normally of uniform size cell-to-cell within a given cyst (Fig. 4G). In *lut* mutants, however, round spermatids typically had four nuclei and one large mitochondrial derivative per cell (Fig. 4H), suggesting complete failure of cytokinesis after meiosis I and II. In contrast, the *rbp4* mutant usually had early round spermatids with the normal one-to-one arrangement of nucleus and mitochondrial derivative, with occasional two-fold larger mitochondrial derivative, indicating much milder cytokinesis defects (Fig. 4I).

### Spermatocyte-specific isoforms of Syp are required for CycB protein expression in mature spermatocytes

To identify factors required for expression of CycB protein in mature spermatocytes, we probed function of predicted RNA-binding proteins expressed in spermatocytes in a small-scale RNAi screen, focusing on transcripts that were low in *bam* mutant testes (spermatogonia) but high in *aly* mutant testes (containing mostly spermatocytes in meiotic arrest), as ascertained by microarray (Chen et al., 2011). RNAi of *syncrip* (*syp*) under control of the Bam-GAL4 driver resulted in spermatocytes that failed to enter the meiotic divisions and arrested, prompting further investigation.

**Fig. 5.**
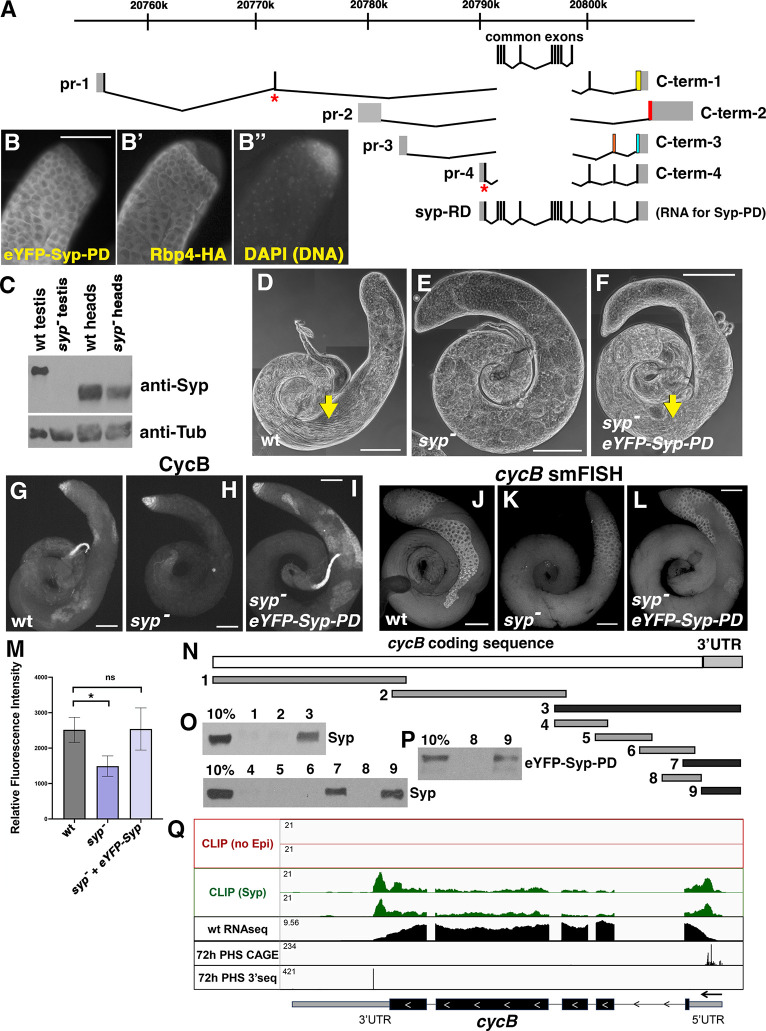
**Loss of function of isoforms of Syp upregulated in testis leads to loss of CycB expression in spermatocytes.** (A) The *syp* genomic locus, showing four promoters and four possible C-terminal ends, based on the FlyBase annotation. Different colors of the C-terminal exons indicate differences in protein isoforms as in Fig. S2E, due to different C-terminal splice forms and/or shifts in reading frame in shared exons. The exons common to all *syp* transcripts are shown above. Asterisks indicate location of the two CRISPR-induced microdeletions that together make the *syp^dub^* allele. The complete *syp-RD* transcript is shown in full. (B-B″) Apical tip of a testis carrying eYFP-Syp-PD and Rbp4-HA transgenes, immunostained with anti-GFP (B) and anti-HA (B′). (B″) DAPI (DNA). (C) Western blots probed with anti-Syp (top) and anti-Tubulin (bottom) of lysate from wild-type (wt) testes, *syp* mutant testes, wild-type heads and *syp* mutant heads The *syp* mutant here and throughout the paper is *syp^dub^/Df*. (D-F) Phase-contrast images of wild-type testes (D), *syp* mutant testes (E) and testes from *syp* mutants carrying *eYFP-Syp-PD* (F). Arrows indicate elongating spermatids. (G-I) Anti-CycB on wild-type testes (G), *syp* mutant testes (H), and testes from syp mutants carrying *eYFP-Syp-PD* (I). (J-L) smFISH with probes against *cycB* on wild-type testes (J), *syp* mutant testes (K) and testes from syp mutants carrying *eYFP-Syp-PD* (L). (M) Quantification of smFISH signal in spermatocytes from the genotypes in J-L. Values represent the mean signal measurement for three testes per genotype. Brackets denote standard deviation. Wild type versus *syp*, *P=0.0190; wild type versus *syp*;*eYFP-Syp-PD*, *P*=0.9526 (ns, not significant) (Welch's *t*-test). (N) Diagram of numbered biotin probes tiled across the *cycB* coding sequence and 3′ UTR showing probes that brought down Syp (black) and probes that did not (gray). (O) Western blots probed with anti-Syp of proteins isolated by biotin pulldown from wild-type testis extract, with probe number indicated. (P) Western blot probed with anti-GFP of biotin pulldowns with probes 8 and 9, from testis extract from eYFP-Syp-expressing flies. (Q) Integrated Genomics Viewer snapshot of the *cycB* locus, with annotated transcript isoform, gene transcribed from right to left. Tracks from top to bottom: CLIP signal from two replicates of wild-type (no-epitope) controls [CLIP (no Epi)]; CLIP signal from two replicates eYFP-Syp [CLIP (Syp)]; wt RNA-seq signal; CAGE signal marking the transcription start site at 72 h post-heat-shock (PHS) (Lu et al., 2020); 3′-seq signal marking where the spermatocyte transcript ends at 72 h PHS (Berry et al., 2022). Note that the 3′ UTR of the transcript isoform expressed in spermatocytes is shorter than the annotated isoform, as indicated by the 3′ seq peak. Scale bars: 100 µm (B-B″,G-L); 200 µm (D-F).

*Drosophila* Syp is the homolog of human SYNCRIP (synaptotagmin binding cytoplasmic RNA interacting protein) (Mizutani et al., 2000; McDermott et al., 2012). Detailed analysis showed that spermatocytes express novel isoforms of Syp RNA and protein. The *syp* locus is complex, with four known promoters, a set of common/shared exons and four possible C-terminal options, generated by alternative splicing (Fig. 5A) that encode predicted proteins with different C-terminal regions (Fig. S2E). Results from RNA-seq, CAGE and RT-PCR analysis indicated that promoters 1 and 4, which produce transcripts with alternate N-terminal coding sequences, fire early in spermatocyte development (Lu et al., 2020 and this study). RT-PCR confirmed that of the four annotated promoters in the *syp* locus, two (promoters 1 and 4 in Fig. 5A) were highly expressed in testis but much less so in head (Fig. S2A). Expression from promoters 1 and 4 was not detected in *bam* testes (0 h PHS), which have spermatogonia but not spermatocytes, but was detected at 24 h PHS and increased by 48 h PHS (Fig. S2A). Promoters 2 and 3 were expressed in testis and head and did not show stage-dependence in the time-course (Fig. S2A, second and third rows).

Of the four 3′ splice options at the *syp* locus, two (C-term-1 and C-term-4) showed robust expression starting soon after the switch from spermatogonia to spermatocytes in the hs-Bam time-course. These two C-terminal options were not detected in head or *bam* mutant testes, but appeared in testis at 24 h PHS and increased by 48 h PHS in the hs-Bam time-course (Fig. S2A, fifth and eighth rows). In contrast, C-term-2 and C-term-3 showed broad expression in testis and head (Fig. S2A, sixth and seventh rows).

The predominant RNA products in testis had the testis-enriched promoters paired with the testis-enriched C-terminal splice forms, or the generally-expressed promoters paired with the generally-expressed splice forms. RT-PCR with pairwise combinations of forward primers corresponding to either promoter 1, 2 or 4 and reverse primers from C-term-1, 2 or 4 showed PCR products from testis extracts for promoter 1 with C-term-1 and -4 only, for promoter-2 with C-term-2 only, and for promoter 4 with all three 3′ splice options (although C-term-2 was the least abundant) (Fig. S2B). These data suggest that the generally-expressed promoter 2 either fires at very low levels in spermatocytes or fires in a different cell type within the testis, such as spermatogonia or somatic cells.

A tagged minigene transgene termed eYFP-Syp-PD (FlyBase annotation), containing a cDNA representing transcript *syp-RD* (promoter 4+C-term-4, Fig. 5A) and 573 bp of sequence directly upstream of the predicted transcription start site (TSS) for promoter 4 drove expression of the eYFP-Syp protein starting in early spermatocytes (Fig. 5B), at about the same time as expression of Rbp4-HA (Fig. 5B′), and not overlapping with the bright DNA signal in the spermatogonial compartment (Fig. 5B”). The protein isoform encoded by the eYFP-Syp-PD transgene was entirely cytoplasmic, consistent with the lack of a predicted nuclear localization sequence (NLS) (Fig. S2E). Reporter transgenes made with either promoter 1 or promoter 4 and with C-term-1 showed nuclear expression in early spermatocytes (consistent with the presence of a predicted NLS encoded by C-term-1), and both nuclear and cytoplasmic localization later (Fig. S2C,D).

Several existing loss-of-function alleles at the *syp* locus that affect all *syp* isoforms result in semi-lethality when placed over a deficiency. To sharply reduce Syp function in the male germline while leaving somatic function of Syp largely intact, we created a doubly-mutated allele of the *syp* locus that disrupted all isoforms emanating from the two predominantly germline promoters. Co-injection of CRISPR RNAs targeting the unique N-terminal coding sequences transcribed from promoters 1 and 4 resulted in creation of a *syp* allele (*syp^dub^*) with a pair of microdeletions, one downstream of each promoter, that resulted in frameshifts and early termination of the corresponding proteins. Flies that were *syp^dub^/Df* (hereafter referred to as *syp* mutants) were viable and female-fertile but male-sterile. Syp protein was not detected by anti-Syp western blot in testes from *syp* mutant flies under conditions where Syp protein was easily detected in wild-type testis extracts (Fig. 5C). While Syp protein was completely gone from the male germline in *syp*, some Syp protein was detected in somatic cells upon immunostaining with anti-Syp of *syp* mutant testes (Fig. S2F,G). Co-staining for Syp (Fig. S2H) and Traffic Jam (Tj) (Fig. S2H′) indicated that the isoforms of Syp present in somatic cyst cells can localize to the nucleus (arrows) as well as the cytoplasm. The Syp protein detected in somatic cyst cells likely was expressed from RNA originating from promoters 2 or 3.

Syp is needed in the male germline for progression through the meiotic divisions and post-meiotic differentiation. In wild-type testes, elongating spermatids were clearly visible (Fig. 5D, arrow), whereas in *syp* mutant testes, no elongating spermatids were observed, and mature-sized late-stage spermatocytes accumulated (Fig. 5E; see also Fig. 7). The eYFP-Syp-PD transgene partially rescued the *syp* phenotype, with elongating spermatids visible (Fig. 5F, arrow).

**Fig. 6.**
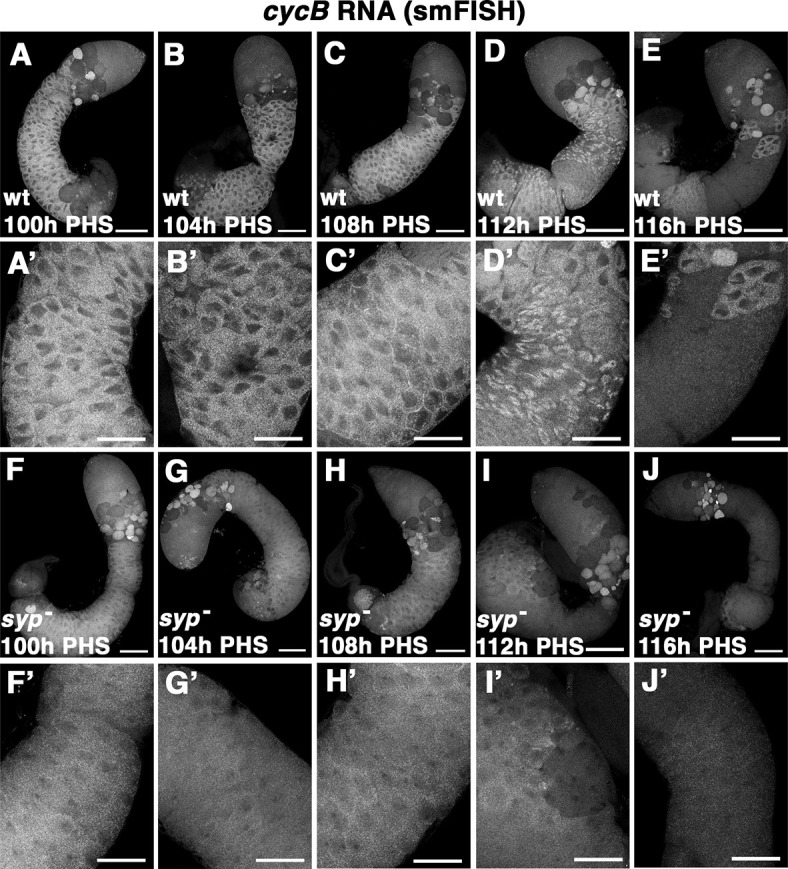
***cycB* RNA levels are lower but detectable through 108 h PHS in *syp* mutants.** (A-J) Testes from time points in the hs-Bam time-course probed for *cycB* RNA by smFISH. (A-E) Wild-type time-course testes. (A′-E′) Higher magnification of spermatocyte regions of A-E. (F-J) *syp* mutant time-course testes. (F′-J′) Higher magnification of spermatocyte regions of F-J. 100 h, 104 h, 108 h, 112 h and 116 h post-heat-shock (PHS) as indicated for both genotypes. Scale bars: 100 µm (A-J); 50 µm (A′-J′).

**Fig. 7.**
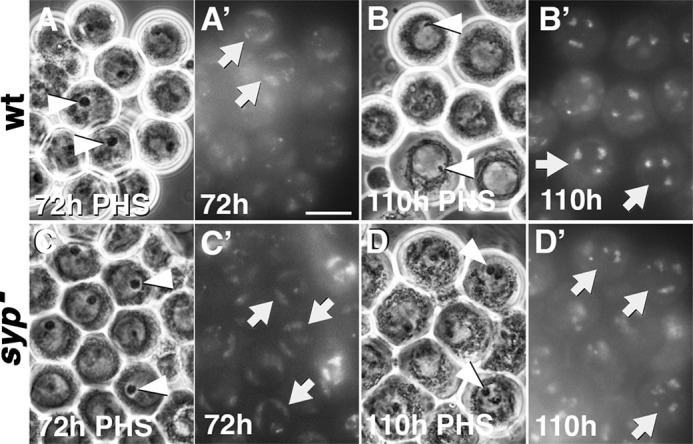
**Spermatocytes mutant for *syp* arrest before prometaphase.** (A-D′) Phase-Hoechst images of spermatocytes in squashed preparations of testes from the hs-Bam time-course on wild type (A-B′) and *syp* mutants (C-D′) at 72 h or 110 h post-heat-shock (PHS) as indicated. (A,B,C,D) Phase-contrast. (A′,B′,C′,D′) Hoechst (DNA). Arrows in A′, B′, C′ and D′ point roughly to the outer edge of the nucleus of interest. Arrows in A′ and C′ indicate nuclei with chunky, crescent-shaped chromosomes; arrows in B′ indicate nuclei with condensed chromosomes; arrows in D′ indicate nuclei with partially condensed chromosomes. Arrowheads in A, C and D indicate intact nucleoli; Arrowheads in B indicate broken-down nucleoli. Scale bar: 25 µm.

Syp function is required for accumulation of CycB in mature spermatocytes. In *syp* mutant testes, CycB protein was expressed normally in spermatogonia but did not reappear in mature spermatocytes (Fig. 5G,H). smFISH with probes against *cycB* indicated that the *cycB* RNA was still present in *syp* mutant spermatocytes, although at lower levels than in wild type (Fig. 5J,K; quantified in M). One copy of the eYFP-Syp-PD transgene was sufficient to rescue expression of CycB protein in mature spermatocytes in a *syp* mutant background (Fig. 5I) as well as restore *cycB* RNA levels in spermatocytes (Fig. 5L; quantified in M). The ability of the exclusively-cytoplasmic eYFP-Syp-PD to rescue CycB protein expression argues that cytoplasmic rather than nuclear functions of Syp are important for CycB protein to be expressed in mature spermatocytes.

Syp protein binds to the 130 nt *cycB* 3′ UTR expressed in spermatocytes. *In vitro* transcribed biotin-labeled probes corresponding to portions of the *cycB* RNA (Fig. 5N) were incubated with testis extracts from wild-type flies and captured with streptavidin beads. Associated proteins were then analyzed by anti-Syp western blot (Fig. 5O). Syp protein associated with probes containing the short spermatocyte 130 nt *cycB* 3′ UTR but not with probes representing the *cycB* protein coding sequences. The eYFP-Syp-PD reporter protein also bound the *cycB* 3′ UTR, in similar biotin pulldown assays from testes from males carrying the eYFP-Syp-PD transgene (Fig. 5P).

Results from ultraviolet (UV) cross-linked and immunoprecipitation (CLIP)-sequencing (easyCLIP, Porter et al., 2021) showed that eYFP-Syp expressed in spermatocytes bound the *cycB* 3′ UTR as well as the 5′ UTR directly *in vivo*. In a pilot experiment, Syp bound an abundant amount of testis RNA. After robust RNAse digestion of UV-crosslinked eYFP-Syp-RNA and ligation of a fluorescent-labeled 5′ adapter, the samples were run on a gel. As expected, eYFP-Syp-RNA+adapter migrated ∼15 kDa higher than free eYFP-Syp (Fig. S3A), indicating that indeed eYFP-Syp directly bound RNA *in vivo*. In a subsequent round of easyCLIP on testes from eYFP-Syp males as well as wild-type testes with no epitope (negative control), sequencing of cDNA libraries made from the recovered RNAs (Fig. S3B) revealed that easyCLIP of eYFP-Syp highly enriched the 3′ UTR of *cycB* and also its 5′ UTR [Fig. 5Q, CLIP (Syp), green]. A histogram of genes expressed in spermatocytes at levels similar to *cycB* showed that the *cycB* RNA bound more Syp than most of the other RNAs (Fig. S3C). The *loopin-1* RNA, which is very highly expressed in spermatocytes, likewise bound more Syp than most of the RNAs in its expression cohort (Fig. S3D). Interestingly, CLIP data showed that Syp could bind spermatocyte RNAs primarily at the 5′ UTR, primarily at the 3′ UTR, at both UTRs or not at all (see Fig. S3E-H).

One possibility is that without Syp bound to the *cycB* 3′ UTR, the *cycB* message might be so destabilized that it is gone by the time spermatocytes are mature enough to translate *cycB*. To address this question, *cycB* RNA levels were assayed in the context of the hs-Bam time-course in both wild type and *syp* mutants. The *cycB* RNA was detected, although at levels considerably lower than wild type, in late-stage and mature spermatocytes in *syp* mutants, past the stage at which *cycB* is normally translated in wild type. In testes from the hs-Bam time-course, smFISH revealed the presence of *cycB* RNA in wild-type mature spermatocytes from 100 h through 112 h PHS (Fig. 6A-D), with *cycB* RNA levels dropping in most cysts by 116 h PHS (Fig. 6E). In *syp* mutant spermatocytes, *cycB* RNA was detected at lower levels than wild type throughout the first three timepoints (Fig. 6F-H), with a drop in *cycB* levels in some cysts at 112 h PHS (Fig. 6I) and in most cysts by 116 h PHS (Fig. 6J).

### Spermatocytes mutant for *syp* arrest before pro-metaphase

Wild-type function of Syp in spermatocytes is required for successful entry into and progression through the meiotic divisions. Wild-type spermatocytes at 72 h PHS in the hs-Bam time-course showed round nucleoli (Fig. 7A, arrowheads), with the main autosomal bivalents appearing as chunky crescents near the nuclear membrane (Fig. 7A′, white arrows). By 110 h PHS most nucleoli in wild-type spermatocytes had shrunk or disappeared and the chromosome bivalents were rounded up, partially condensed and had moved away from the nuclear periphery (Fig. 7B′, white arrows) in advance of congressing to the center of the nucleus for metaphase (a stage documented in wild-type testes by Baker et al., 2015; Baker and Fuller, 2007; Lin et al., 1996). Spermatocytes mutant for *syp* showed intact nucleoli (Fig. 7C, arrowheads) and normal chunky crescent-shaped bivalents next to the nuclear envelope at 72 h PHS (Fig. 7C′) but showed only partially-condensed chromosomes at 110 h PHS (Fig. 7D′, arrows) and did not show nucleolar breakdown (Fig. 7D, arrowheads). Metaphase figures (chromosomes in a tight knot at the center of the nucleus) were never observed in *syp* mutants. Unlike *twe* and *boule* mutants, which skip the meiotic divisions and complete a convincing (*twe*) or modest (*boule*) amount of post-meiotic differentiation (Alphey et al., 1992; Eberhart et al., 1996), *syp* mutant germ cells did not show any signs of post-meiotic differentiation, such as formation of the nebenkern, which normally takes place in round spermatids (as seen in wild type in Fig. 4D).

### Syp binds to Fest and interacts with Rbp4 and Lut dependent on Fest

**Fig. 8.**
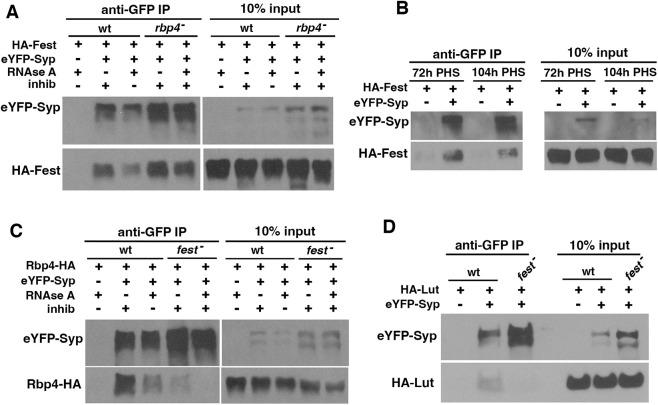
**Syp interacts with Fest and co-immunoprecipitates with Rbp4 and Lut in the presence of Fest.** (A) Western blots probed with anti-GFP or anti-HA of anti-GFP immunoprecipitates from testes expressing eYFP-Syp and HA-Fest, with either RNAse A or RNAse inhibitor, as indicated. Negative control: HA-Fest alone. Flies were either wild type (wt; first three lanes) or mutant for *rbp4* (lanes 4 and 5). (B) Western blots probed with anti-GFP or anti-HA of anti-GFP immunoprecipitates from hs-Bam time-course testes expressing eYFP-Syp and HA-Fest, collected at 72 h and 104 h post-heat-shock (PHS), as indicated. All samples included RNAse A. Negative controls: HA-Fest only. (C) Western blots probed with anti-GFP or anti-HA of anti-GFP immunoprecipitations from testes expressing eYFP-Syp and Rbp4-HA, with either RNAse A or RNAse inhibitor, as indicated. Negative control: Rbp4-HA alone. Flies were wt (first three lanes) or mutant for *fest* (lanes 4 and 5). (D) Western blots probed with anti-GFP or anti-HA of anti-GFP immunoprecipitates from testes also expressing eYFP-Syp and HA-Lut. All samples included RNAse A. Flies were wt or mutant for *fest*, as indicated. Negative control: HA-Lut alone.

The cytoplasmic isoform of the Syp protein encoded by Syp-PD co-immunoprecipitated Fest, in an interaction that did not require Rbp4. Immunoprecipitation of eYFP-Syp with anti-GFP from testis extracts brought down HA-Fest (Fig. 8A). This interaction remained strong in the *rbp4* mutant background, suggesting that Fest and Syp do not require Rbp4 for their interaction. The translation of *cycB* in mature spermatocytes is not likely due to dissociation of Syp from Fest, as immunoprecipitation of eYFP-Syp brought down HA-Fest at both 72 h and 104 h PHS (Fig. 8B).

Rbp4-HA also co-immunoprecipitated with eYFP-Syp, but this interaction required Fest. Immunoprecipitation with anti-GFP from testes expressing both eYFP-Syp and Rbp4-HA brought down Rbp4-HA (Fig. 8C), but this interaction was reduced in the presence of RNAse and in a *fest* mutant background (Fig. 8C), suggesting that the physical interaction between Rbp4 and Syp may be mediated by their individual interactions with Fest and with RNA. Similarly, HA-Lut co-immunoprecipitated with eYFP-Syp in the presence but not absence of Fest (Fig. 8D), in the presence of RNAse. Together, these data suggested that, in addition to being bound to the *cycB* 3′ UTR, Syp also associates with Rbp4, Fest and Lut via its binding with Fest.

### Syp is not required for *cycB* translation in young spermatocytes lacking Rbp4 function

**Fig. 9.**
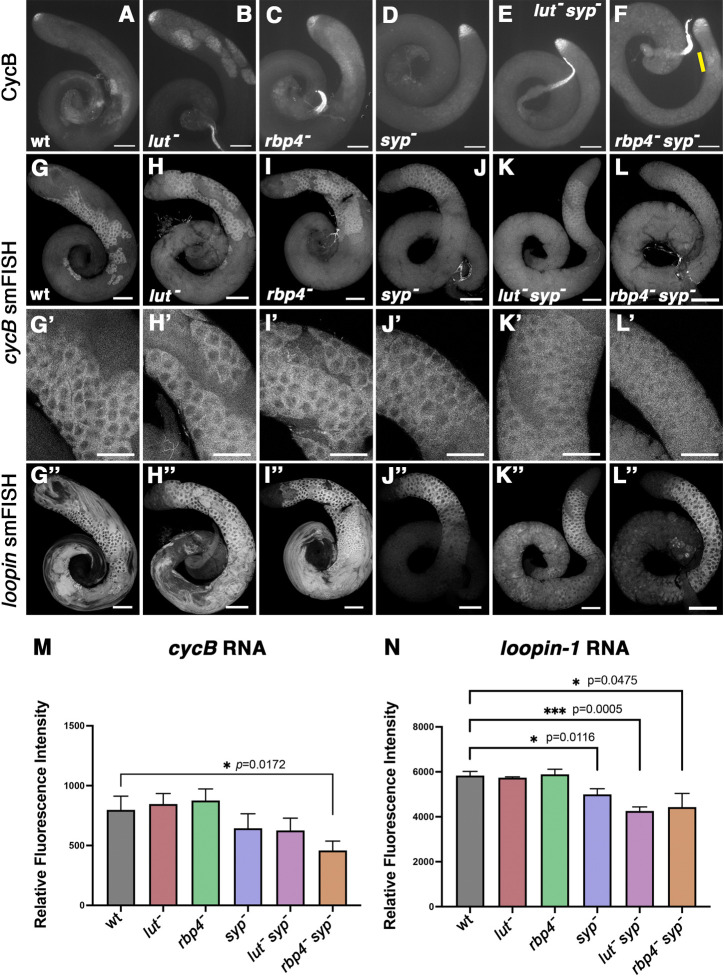
**In the absence of Rbp4, Syp is not required for *cycB* translation in mid-stage spermatocytes.** (A-F) Immunofluorescence images of whole testes stained with anti-CycB showing wild type (wt; A) and *lut* (B), *rbp4* (C), *syp* (D), *lut syp* (E) and *rbp4 syp* (F) mutants. Yellow bar in F highlights a puff of CycB expression. (G-L) Whole testes probed for *cycB* RNA by smFISH showing wild type (wt; G) and *lut* (H), *rbp4* (I), *syp* (J), *lut syp* (K) and *rbp4 syp* (L) mutants. (G′-L′) High magnification views of spermatocyte regions in (G-L). (G″-L″) The same testes probed for *loopin-1* RNA by smFISH. (M,N) Quantification of *cycB* (M) and *loopin-1* (N) smFISH signal in wt and *lut*, *rbp4*, *syp*, *lut syp* and *rbp4 syp* mutant spermatocyte regions. Values represent the mean signal measurement for three testes per genotype. Error bars denote standard deviation. *P*-values for mutant versus wild type shown where *P*<0.05 (Welch's *t*-test). Scale bars: 100 µm (A-L;G″-L″); 50 µm (G′-L′).

Analysis of CycB protein expression in testes from *rbp4 syp* double-mutant flies revealed that Syp was not required for CycB protein expression in young spermatocytes in the absence of Rbp4. Testes from wild-type, single (*lut*, *rbp4* and *syp*) and double (*lut syp* and *rbp4 syp*) mutants were stained using anti-CycB antibodies (Fig. 9A-F). In testes from *rbp4 syp* double-mutant flies, a puff of CycB protein expression was detected (Fig. 9F, bar), arguing against the possibility that Rbp4 acts by blocking the ability of Syp to activate translation of *cycB* in immature spermatocytes. In *lut syp* double mutants, in contrast, CycB was not detected in spermatocytes (Fig. 9E) – comparable with testes mutant for *syp* alone (Fig. 9D). The result that Syp is still required for CycB expression in late-stage spermatocytes in the absence of Lut indicates that Syp does not promote *cycB* translation by blocking repressive action by Lut.

smFISH with *cycB* probes on an equivalent set of wild-type and mutant testes (Fig. 9G-L; high magnification in G′-L′) showed that *cycB* RNA levels were low in the *rbp4 syp* double mutant compared with wild type (Fig. 9G,L; quantified in M), suggesting that Rbp4 contributes a modest amount to the stability of the *cycB* RNA in the absence of Syp. The fact that onset of premature CycB expression in *rbp4 syp* double mutants appeared to be similar to that in *rbp4* mutants alone, but the CycB protein did not perdure as long in *rbp4 syp* double mutants as in *rbp4* mutants, could be due to a continued decrease in *cycB* RNA abundance as spermatocytes mature and/or changes in the translational environment in later spermatocyte stages. Parallel smFISH using probes against the *loopin-1* RNA (Fig. 9G″-L″; quantified in N) revealed that Syp may help stabilize the *loopin-1* RNA as well as *cycB*, consistent with the results from the Syp easyCLIP experiment described above indicating that *loopin-1* may also be a target of Syp (Fig. S3D,E).

## DISCUSSION

Our work reveals that during meiotic prophase the male germline developmental program controls timing of expression of the core G2 cell cycle regulator CycB through cell-type-specific translational repressors as well as a binding partner that stabilizes the *cycB* RNA and may also activate its translation. Three of the earliest genes newly expressed when *Drosophila* spermatocytes enter meiotic prophase encode interacting proteins that act to prevent premature expression of the CycB protein. The Rbp4 protein is required to block translation of the *cycB* RNA in mid-stage spermatocytes and Lut is required to block translation of *cycB* in late-stage, not-quite-mature spermatocytes. Thus, when *cycB* transcription resumes from the spermatocyte-specific promoter, the newly-expressed transcripts enter a cytoplasm already prepared for their translational repression. Complementing this cell-type-specific repression of translation, spermatocytes also express a cytoplasmically localized protein isoform of the SYNCRIP homolog Syp, which binds strongly to the *cycB* 5′ and 3′ UTRs and is required for the normal ability of mature spermatocytes to express CycB protein immediately before entry into the meiotic divisions.

Loss of function of either Lut or Rbp4 resulted in premature entry into the meiotic divisions and early progression to haploid round spermatids, with a difference of about 6-8 h from wild-type controls. Our results indicate that there is a narrow window of opportunity in late spermatocytes where premature CycB expression may help promote early meiotic entry. In contrast, expression of CycB at 54 h PHS in an *rbp4* mutant did not cause immediate entry into the meiotic divisions. It is likely that spermatocytes require additional crucial core G2 cell cycle regulators to undergo the G2/M transition of meiosis I. If such additional regulators only become active by the stage equivalent to 90-96 h PHS, premature expression of CycB may be inadequate to help drive the G2/M transition before that time.

The premature meiotic entry in a *lut* mutant occurred about 2 h earlier than in an *rbp4* mutant (Fig. 4E), and the cytokinesis defects were more severe (Fig. 4H). Rbp4 likely has additional target RNAs other than *cycB*, as Haynes and colleagues noted that several proteins were upregulated in the absence of Rbp4 function, based on their analysis of *rbp4 boule* versus *boule* testis extracts by 2D gel (Haynes et al., 1997). We suspect that Lut also has additional targets – not regulated by Rbp4 – misregulation of which may contribute to the stronger *lut* mutant phenotype.

The difference in timing of CycB protein expression in *rbp4* versus *lut* mutants points to two distinct states in the regulation of *cycB* translation. Rbp4 is required for repression of *cycB* translation in mid-stage spermatocytes (54 h-94 h PHS), whereas Lut function is not required to prevent CycB protein accumulation at this stage. In late-stage spermatocytes (94 h-102 h PHS), in contrast, function of Lut is required for translational repression of *cycB* and, in the absence of Lut, Rbp4 function is either off or not sufficient to repress *cycB* translation.

Rbp4, Lut and Syp appear to interact with each other, anchored by Fest (Figs 2 and 8). As we have yet to detect changes in the individual interactions in early versus late spermatocytes, it remains a mystery as to how translational repression by the complex is eventually relieved in mature spermatocytes preparing for the G2/M transition of meiosis I. However, using results from epistasis tests (Fig. 9), we can postulate possible regulatory relationships among the interacting proteins in mid-stage, late-stage and mature spermatocytes. The three models presented here are not meant to rule out other possibilities, but each one is compatible with the data from single mutants, double mutants and our time-course. In particular, expression of CycB protein in young *rbp4 syp* double mutant spermatocytes indicates that Rbp4 does not act upstream of Syp (for example, by blocking *cycB* translation by inhibiting action of Syp). Likewise, lack of detectable CycB protein in *lut syp* double mutant spermatocytes would suggest that Syp does not act upstream of Lut (for example, allowing translation by turning off repression by Lut).

**Fig. 10.**
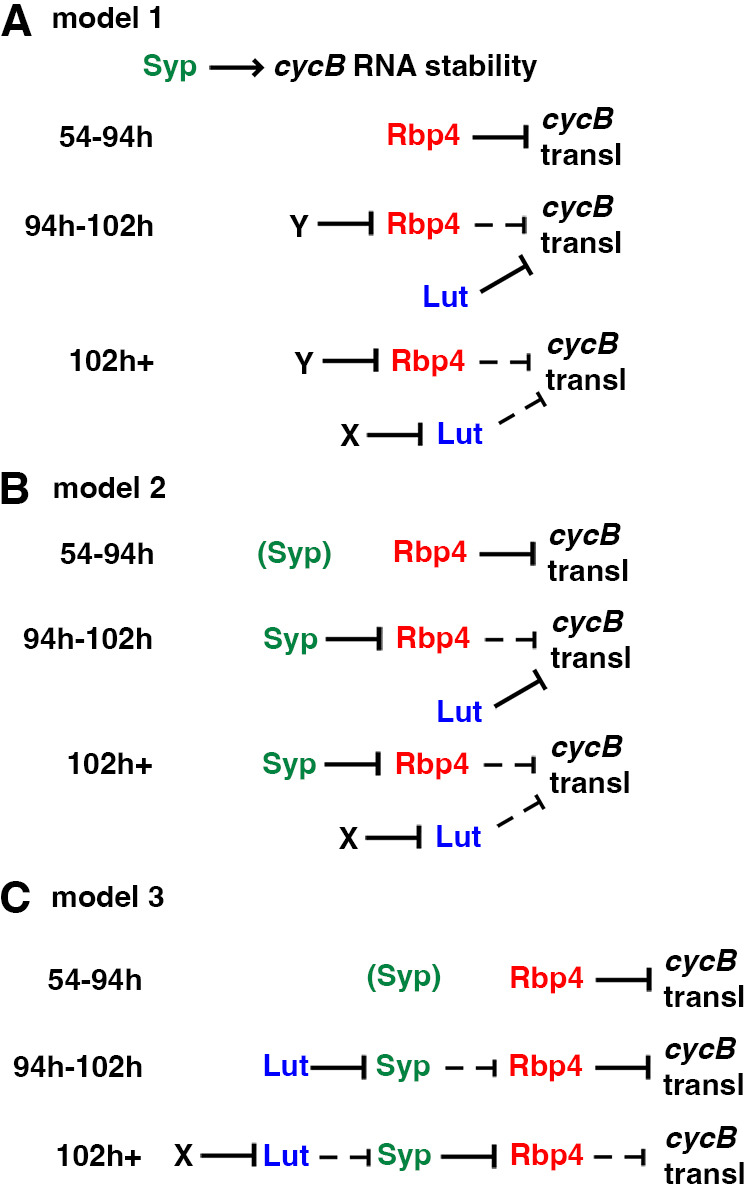
**Three models for Lut, Syp and Rbp4 function in regulating *cycB* translation and stability.** Solid lines indicate active promotion or repression; dotted lines indicate abrogated function. Not shown, but contributing to all three models: Syp and Rbp4 act in parallel to stabilize the *cycB* RNA. See Discussion.

The first model (Fig. 10A) postulates that: (1) Rbp4 represses translation of *cycB* in mid-stage spermatocytes (∼54-94 h PHS), subject to reversal by factor Y; (2) Lut represses translation of *cycB* in late-stage spermatocytes (∼94-102 h PHS), subject to reversal by factor X in mature spermatocytes (∼102 h+PHS); and (3) Syp functions primarily to stabilize *cycB* RNA and maintain it at sufficiently high levels to produce detectable amounts of CycB protein in mature spermatocytes. Levels of *cycB* RNA were clearly reduced in the absence of Syp (Figs 5, 6 and 9). One possible explanation is that occupancy of the short *cycB* 3′ UTR by Syp protein blocks binding of other proteins that could bind to the 3′ UTR and recruit the deadenylase complex to the target RNA (Fukushima et al., 2019; Arvola et al., 2020; Semotok et al., 2005). As noted above, *cycB* RNA was detected at 104 and 108 h PHS in the *syp* mutant (Fig. 6), albeit at lower than normal levels. However, the baseline translational environment at that stage may be less permissive than the environment in mid-stage spermatocytes, such as those that express CycB protein in the *rbp4 syp* double mutant (Fig. 9).

The second model (Fig. 10B) presents the possibility that Syp could be factor Y, suppressing Rbp4 function in maturing spermatocytes, while still acting in parallel with Rbp4 to stabilize the *cycB* RNA. In this model, Syp present in mid-stage spermatocytes is not capable of blocking translational repression by Rbp4 until late-stage spermatocytes, setting up the requirement for Lut in the 94-102 h PHS window as the last bastion of *cycB* translational repression, until Lut function itself is counteracted at 102 h PHS by factor X.

In the third model (Fig. 10C), Lut, Syp and Rbp4 function in a sequential repressive cascade. In mid-stage spermatocytes, as in model 2, Rbp4 represses *cycB* translation, with Syp present in the complex but not capable of interfering with Rbp4 function. In late-stage spermatocytes, Syp is capable of blocking Rbp4 repressive activity, but is prevented from doing so by Lut. Thus, loss of function of Lut allows *cycB* translation in this late-stage window. In mature spermatocytes, factor X counteracts Lut function, releasing Syp to antagonize Rbp4 function, thus allowing *cycB* translation.

Consistent with these models, both *Drosophila* Syp and mammalian SYNCRIP homologs have been implicated in regulation of RNA stability, localization and translation. In *Drosophila*, Syp has been shown to regulate developmental and cell fate decisions in a diverse set of cell types and tissues, with specific RNA targets identified in some cases. Syp functions in the oocyte to promote dorso-anterior localization of the *gurken* RNA, binding the *gurken* localization signal (McDermott et al., 2012). In the larval neuromuscular junction, Syp acts post-synaptically to block synaptic overgrowth, binding multiple target RNAs (McDermott et al., 2014). Syp binds the *MSP-300* RNA, co-localizes with it *in vivo* to ribosome-dense granules (Titlow et al., 2020) and promotes translation of *MSP-300* in a neural-activity dependent manner. In the *Drosophila* mushroom body, Syp and another RNA-binding protein, Imp, appear in opposing temporal gradients and regulate cell fate decisions (γ, α′/β′ or α/β neurons), with Syp antagonizing and Imp promoting expression of Chinmo protein without either factor affecting *chinmo* RNA abundance (Liu et al., 2015). Syp promotes neuroblast decommissioning by upregulating expression of the Prospero protein (Yang et al., 2017) via stabilization of the long form of the *prospero* RNA expressed in larvae (Samuels et al., 2020). From this extensive literature, it is clear that Syp can both stabilize RNAs and also have repressive or activating effects on translation of specific target RNAs, sometimes even in a single cell type (McDermott et al., 2014).

## MATERIALS AND METHODS

### Fly genetics and husbandry

Flies were raised on dextrose/cornmeal food at 20°C for stocks and 25°C for crosses. The *rbp4* mutant flies were *rbp4^LL06910^/Df(3R)Exel6169*. *Df(3R)Exel6169* is from Bloomington *Drosophila* Stock Center (BDSC, #7648) and the *rbp4^LL06910^* allele was from the *Drosophila* Genomics Resource Center (DGRC, #141934). The *lut^1^* and *syp^dub^* alleles were generated by CRISPR as described below. The *lut* mutant flies were *lut^1^/Df(2R)BSC131*. *Df(2R)BSC131* is from BDSC (#9296). The *syp* mutant flies were *syp^dub^/Df(3R)BSC141*, with *Df(3R)BSC141* from BDSC (#9501). For the hs-Bam time-course, ∼40 *w*; *hs:Bam-HA*/*CyO*; *bam*^Δ*86*^/*TM3* virgin females were mated to *w*;; *bam^1^*/*TM6B* males for wild-type controls. To generate *lut* mutants in the time-course, *w*; *hs:Bam-HA*, *lut^1^*/*CyO*; *bam*^Δ*86*^/*TM3* females were mated with *w*; *Df(2R)BSC131*; *bam^1^*/*TM6B* males. For *rbp4* mutants in the time-course, *w*; *hs:Bam-HA*/*CyO*; *Df(3R)Exel6169*, *bam*^Δ*86*^/*TM3* females were mated with *w*;; *rbp4^LL06910^*, *bam^1^*/*TM6B* males. For *syp* mutants in the time-course: *w*; *hs:Bam-HA*/*CyO*; *Df(3R)BSC141*, *bam*^Δ*86*^/*TM3* females were mated with *w*;; *syp^dub^*, *bam^1^*/*TM6B* males.

### Hs-Bam time-course

For the hs-Bam time-course, fly bottles (plastic) containing mid- and late-stage pupae were weighted down and placed in a 37°C water bath with water nearly up to the lip of the bottle. After 30 min the bottles were cleared of any adults that may have eclosed, labeled with date and time of heat shock, and returned to 25°C for the designated time post heat shock. The start of the hours-post-heat-shock clock was set to the end of the 30-min 37°C incubation. Note that time-course testes regain a population of *bam* mutant spermatogonia at the apical end of the testis during the incubation at 25°C after heat shock, due to mitotic proliferation of *bam* mutant early germ cells that avoided forced differentiation. Within a heat-shock time-course testis, differentiating spermatocytes are only meta-synchronous: there is a distribution of cysts at various stages, visible when scoring short-duration events such as the meiotic divisions. This intra-testis asynchrony was observed in all three genotypes scored for Fig. 4, where we were scoring the leading edge of the distribution of cysts entering and exiting meiotic division. An *n* of 100 per timepoint per genotype was chosen as a sufficiently large sample to get an accurate result, given the meta-synchrony of meiotic entry.

### CRISPR alleles

Microdeletions were generated by CRISPR using the technique described in Bassett et al. (2013). Genomic target sites were chosen early enough in the coding sequence to cause a significant disruption to the encoded protein, and containing the sequence GN[N_18_]NGG in either direction. This sequence was then incorporated into the middle of a forward primer after changing the first N to G, and removing NGG, to produce the following oligo: GAAATTAATACGACTCACTATA**GG(N_18_)**GTTTTAGAGCTAGAAATAGC, containing a T7 promoter (underlined) followed by the adjusted gene-specific sequences (bold). Template-less PCR was performed with this primer and a common reverse primer (AAAAGCACCGACTCGGTGCCACTTTTTCAAGTTGATAACGGACTAGCCTTATTTTAACTTGCTATTTCTAGCTCTAAAAC) as described previously (Bassett et al., 2013), then cleaned up using a PCR purification kit. We used 250 ng of PCR product as a template for an *in vitro* transcription reaction (MEGAscript T7 kit), incubated at 37°C for 2 h. After treatment with DNAse for 15 min, the resulting RNA was purified by Trizol prep. For each target, the guide RNA was then injected into Act5-Cas9 embryos at a concentration of 100 ng/µl. Surviving adults were individually crossed to the deficiency line uncovering the gene of interest, and the resulting progeny were screened for the phenotypes observed previously via testis-specific RNAi knockdown [Vienna *Drosophila* Resource Center (VDRC) #33011 for *syp* and VDRC #29664 for *lut*]. The gene-specific GN[N_18_]NGG sequence for *lut* was GGGCCAGCAGGGATTCCATTTGG and the resulting microdeletion in *lut^1^* was GGGCCAGCAGGGxxxxxATTTGG, deleting nucleotides 136-140 of the *lut* protein coding sequence. This resulted in a frameshift just after the codon for Gly45, followed by three novel residues (I, W, G), then a stop codon, whereas wild-type Lut is 132 amino acids. For *syp*, the gene-specific sections of the guide RNAs were GTACCCGTTATCAAGCCCATTGG (downstream of promoter 1) and GGAAGCCTCCAAAGTGCAGAAGG (downstream of promoter 4), and their co-injection generated the *syp^dub^* allele, which contains two microdeletions in tandem: GTACCCGTTATCAAGCxCATTGG (pr-1) and GGAAGCCTCCAAAxxxxxxxxGG (pr-4). The mutated coding sequence downstream of promoter 1 encodes the first 26 correct amino acids, 82 aberrant residues and a stop codon, whereas the mutated coding sequence downstream of promoter 4 encodes the first nine correct amino acids, one aberrant residue and a stop codon.

### Transgenes

The transgenes described below are shown in Fig. S4. The eYFP-Syp-PD reporter, built in pBlueScript-KS and then moved into the *Not*I/*Spe*I sites of pCaSpeR4, consisted of: *Not*I – *syp* promoter-4 (573 bp, directly 5′ of FlyBase-annotated *syp-RD* start site) and the *syp-RD* 5′ UTR (223 bp) – *Xba*I/*Spe*I [non-recleavable] – *eYFP* coding sequence (717 bp) – *Xba*I – *syp-RD* coding sequence (2124 bp) – *Xba*I – *syp-RD* 3′ UTR and 3′ downstream genomic sequence (811 bp combined) – *Spe*I. The eYFP-Syp-PT and eYFP-Syp-PV reporters were built in pBS-KS and moved into the *Not*I/*Spe*I sites of pCaSpeR4 to which the SV40 terminator had been added. Note that the promoter for *syp-RD* and *syp-RV* is the same, and the C-terminal coding sequence and 3′ UTR are identical for *syp-PV* and *syp-PT*. The eYFP-Syp-PV transgene consisted of: *Not*I – *syp* promoter-4 (573 bp, directly 5′ of annotated *syp-RD/RV* start site) and the *syp-RD/RV* 5′ UTR (223 bp) – *Xba*I/*Spe*I [non-recleavable] – *eYFP* coding sequence (717 bp) – *Xba*I – *syp-RV* coding sequence (2037 bp) – *Xba*I – *syp-RT/RV* 3′ UTR and 3′ downstream genomic sequence (804 bp combined) – *Spe*I. The eYFP-Syp-PT transgene consisted of: *Not*I – *syp* promoter-1 (494 bp, directly 5′ of annotated *syp-RT* start site) and the *syp-RT* 5′ UTR (508 bp) – *Xba*I/*Spe*I [non-recleavable] – *eYFP* coding sequence (717 bp) – *Xba*I – *syp-RT* coding sequence (2013 bp) – *Xba*I – *syp-RT/RV* 3′UTR and 3′ downstream genomic sequence (804 bp combined) – *Spe*I.

The V5-Lut and HA-Lut reporters, built in pBS-KS and moved to the *Not*I/*Xho*I sites of pCaSpeR4, consisted of: *Not*I – 232 bp 5′ genomic and 199 bp 5′ UTR of *lut* – *Xba*I – V5 or 3×HA coding sequence (42 bp and 126 bp, respectively) – *Eco*RI – *lut* coding sequence plus an intron (467 bp total), *lut* 3′ UTR and 3′ downstream genomic sequence (534 bp combined) – *Xho*I. HA-Lut rescued the fertility defects in *lut^1^/Df* males in a small-scale assay: in single male crosses, 6/6 HA-Lut/+; *lut^1^/Df* males fathered larvae versus 0/6 *lut^1^/Df* males and 6/6 wild-type males.

HA-Fest, like eYFP-Fest (Baker et al., 2015), was built in pBS and then moved into the *Xba*I/*Xho*I sites of pCaSpeR4 to which the SV40 terminator had been added. The plasmid contained: *Xba*I – the *fest* promoter (590 bp, directly 5′ of the annotated TSS) and the *fest* 5′ UTR (275 bp) – *Spe*I – 3×HA coding sequence (126 bp) – *Sma*I – the *fest* coding sequence (1542 bp) – *Eco*RI – the *fest* 3′ UTR and 3′ downstream genomic sequence (1255 bp total) – *Eco*RI-*Xho*I – *SV40* terminator – *Sal*I/*Xho*I [non-recleavable]. Transgenic flies were generated by microinjection either at BestGene (eYFP-Syp and HA-Fest) or in-house (the remainder).

Coding sequences for *syp* and *fest*, as well as the 5′ UTR of *syp-RT*, were amplified from cDNA generated from wild-type testis RNA. All other gene-specific sequences listed above were amplified from wild-type genomic DNA.

### RT-PCR

RNA was collected from wild-type testes and heads, as well as hs-Bam time-course testes from 0, 24 and 48 h post-heat-shock. Reverse transcription with oligo-dT primer and Ready-to-Go You-Prime First-Strand Beads (Cytiva, #27926401) was performed on 1 µg of each RNA sample. PCR reactions were carried out in a 25 µl volume with 2 µl cDNA each. BioMix Red (Bioline, #BIO-25006) was used for all reactions except that which amplified C-term-4, which is GC-rich and required MyTaq (Bioline, #BIO-21105). The amplification ran for 30 cycles (Fig. S2A) or 35 cycles (Fig. S2B). Primers for Fig. S2A: the common reverse primer for assaying promoters was 5′-GTTCTCGAATAGCGGAATCAG-3′. Forward primers: promoter 1, 5′-GTCAGAAACACTCGCATGCAAA-3′; promoter 2, 5′-ACTCACTTGGATACACAGCG-3′; promoter 3, 5′-TGTGCAACGCCGAGCAGAGT-3′; promoter 4, 5′-GATGGAAGCCTCCAAAGTGC-3′. Common forward primer (for use on C-term 1, 2 and 3): 5′-CAGGACGTCTCAGAGGATAA-3′. Forward primer for use on C-term 4: 5′-GTGAATACGACTACTTTTACGAC-3′. Reverse primers: C-term-1, 5′-TGCAGCTCCAGCATAAGGCT-3′; C-term-2, 5′-TACCGACTACGTATTCACGG-3′; C-term-3, 5′-CGACCAACTCCCGTATTCAC-3′; C-term-4, 5′-GCTCCAGCTGGTAAATTTTGT-3′. For the *GAPDH2* controls, forward and reverse primers were 5′-CCGTTCATGCCACCACCGCT-3′ and 5′-GCCACGTCCATCACGCCACA-3′, respectively. For Fig. S2B, the relevant isoform-specific forward and reverse primers listed above were used for promoters 1, 2, and 4 and C-terms 1 and 2, and a new primer was used as a reverse primer for C-term 4: 5′-ACGACCAACTCCCATAAGGCT-3′.

### Biotin pulldowns

Probes were cloned into pBS-KS in the orientation *Spe*I 5′→3′ *Bam*HI, with the T7 promoter 5′ of the *Spe*I site. The following probes were used, with the nt numbers corresponding to the 1593 nt protein coding sequence and the short spermatocyte 3′ UTR (130 nt) of *cycB-RA*, starting with the first base of the coding sequence (total, 1723 nt): #1, 1-631; #2, 589-1187; #3, 1117-1723; #4, 1117-1292; #5, 1253-1440; #6, 1392-1574; #7, 1534-1723; #8, 1463-1593; #9, 1594-1723. Plasmids containing the probe templates were linearized with *Bam*HI for 2 h, and then run on a gel and purified using the Zymogen gel cleanup kit. For *in vitro* transcription, T7 polymerase and biotin labeling mix (Roche, #11685597910) were used according to the package directions. After 2 h of transcription at 37°C, 2 µl DNAse was added and reactions were returned to 37°C for an additional 30 min. Probes were cleaned of unincorporated nucleotides using NucAway columns (Thermo Fisher Scientific, #10070). Fifty pairs of testes per sample (plus five pairs per genotype as input) were mechanically lysed in [100 µl/sample+10 µl extra] lysis buffer [100 mM NaCl, 50 mM Tris, 4 mM EDTA, 1% NP40, 1 µl/ml SUPERaseIN (Thermo Fisher Scientific, #AM2694), and 1× cOmplete Protease Inhibitor (Roche, #11836153001)] with a 1 cc syringe and 25×5/8 needle, rocked at 4°C for 30 min and centrifuged at full speed (21,000 ***g***) for 3 min at 4°C to pellet insoluble material. While the lysate was rocking, 50 µl suspended streptavidin MagneSphere beads (Promega, #Z5481) per sample were washed 3×5 min in 0.5× SSC, with a 6 Tube Magnetic Stand (Thermo Fisher Scientific, #AM10055) used to secure beads between washes. Then 10% input (10 µl lysate) was removed for each genotype, supplemented with Laemmli sample buffer to 1×, and boiled; the remaining 100 µl lysate per sample was pre-cleared by incubating with just-prepped beads for 30 min at room temperature (RT). Pre-cleared lysate was removed from those beads and transferred into fresh tubes in 100 µl aliquots (one per probe), into each of which 500 ng of a single biotin-labeled probe was added. Samples were rocked for 30 min at RT, after which the lysate/probe mixture was incubated with fresh 50 µl streptavidin beads for 30 min at RT with rocking. The beads were washed 5×10 min in 1 ml cold lysis buffer at 4°C with rocking. Laemmli sample buffer was added to a concentration of 1× before the samples were boiled. After boiling, samples (including beads) were centrifuged briefly (15 s at 21,000 ***g***), and the supernatant was loaded onto a protein gel and analyzed by western blot.

### Immunoprecipitations

Protein A Dynabeads (Thermo Fisher Scientific, #10002D) were used with rabbit anti-HA (Cell Signaling Technology, #3724S), and pan-mouse IgG Dynabeads (Thermo Fisher Scientific, #11041) were used for anti-GFP (mouse, Millipore Sigma #11814460001) and anti-V5 (mouse, Thermo-Fisher Scientific, #R960-25) (2 µl undiluted antibody was used per 30 µl of suspended beads per immunoprecipitation). The 6 Tube Magnetic Stand from Thermo Fisher Scientific was used, as above, to segregate the beads from liquid between washes during antibody conjugation and immunoprecipitations. We washed 50 µl of suspended beads per sample twice in 1 ml 3% bovine serum albumin (BSA)/PBSTw (PBS+0.1% Tween), brought back up to the original volume (50 µl per sample) and split into two aliquots: 20 µl/sample to be used in the pre-clear step, and 30 µl/sample to be used for antibody conjugation and subsequent immunoprecipitation. To conjugate antibody to beads, excess BSA wash was removed from the suspended beads, and beads were incubated with lysis buffer and antibody in a ratio of 30 µl (suspended) beads:200 µl lysis buffer (see below, but without protease inhibitor):2 µl of antibody per immunoprecipitation. For example, for five immunoprecipitation reactions: 10 µl antibody, 1 ml lysis buffer, 150 µl suspended beads. Beads were incubated with antibody for 1 h at room temperature, then split into individual aliquots (one per immunoprecipitation) and washed 3×5 min in 1 ml 0.2 M triethanolamine (pH 8.2). Beads were then treated for 30 min at RT with triethanolamine to which dimethylpimelimidate had been freshly added to a final concentration of 5.4 mg/ml. After a 15-min wash in 1 ml 50 mM Tris (pH 7.5) and a final wash with 1 ml PBSTw, beads could be stored at 4°C for 1-2 days before use. On the day of the immunoprecipitation, beads were stripped of non-covalently bound antibody via two quick washes in 1 ml 100 mM glycine (pH 2.5). After a wash with PBSTw, they were blocked in 1 ml 10% BSA/50 mM Tris for 1 h before a 5 s rinse in PBSTw.

Testes (55 pairs per sample unless noted otherwise) were mechanically lysed in 220 µl cold lysis buffer (135 mM NaCl, 20 mM Tris, 10 mM EDTA, 1% NP40, 10% glycerol, 1× cOmplete protease inhibitor) per sample with a 1 cc syringe and 25×5/8 needle at 4°C. Samples were then incubated for 20 min on a rocker at 4°C before samples were centrifuged at full speed (21,000 ***g***) for 3 min to pellet insoluble material. The supernatant was collected and split evenly if needed (for ‘+ RNAse’ versus ‘+ inhibitor’). For ‘+ RNAse’ samples, RNAse A (Thermo Fisher Scientific, #EN0531) was added to a final concentration of 100 µg/ml; for ‘+ inhibitor’ samples, SUPERasin was added to a final concentration of 1 U/µl. To pre-clear the lysate, each sample was then transferred to a fresh tube with 20 µl suspended BSA-washed beads (from above; excess BSA wash removed just before using) and incubated at 4°C for 45 min with rocking. Then, 20 µl of lysate was removed from each sample to serve as 10% input, to which 5 µl 5× Laemmli sample buffer was added before boiling. The remaining 200 µl lysate for each sample was transferred to a fresh tube containing antibody-conjugated beads for the immunoprecipitation step (3-4 h at 4°C, rocking). Beads were then washed 2×10 min in 1 ml cold lysis buffer at 4°C while rocking. Bound proteins were eluted from the beads in 40 µl elution buffer [10 mM EDTA, 50 mM Tris (pH 8), 1% SDS, and 1× cOmplete protease inhibitor] at 70°C for 30 min with frequent mixing. The resulting 40 µl eluate was transferred to a fresh tube, and 10 µl 5× Laemmli sample buffer was added. Samples were boiled for 10 min and frozen until analysis by western blot. For Fig. 8A, 77 pairs of *rbp4* testes were used (versus 55 for wild type), as *rbp4* testes appear slightly smaller than wild type. For Fig. 8C,D, 330 pairs of *fest* testes were used, as they are significantly smaller than wild type.

For the IP-MS experiments, beads were prepared as above except with 3 µl anti-GFP antibody in 200 µl for 50 µl suspended beads per immunoprecipitation, with two immunoprecipitation tubes per genotype. One thousand testis pairs were dissected per genotype, lysed in 900 µl lysis buffer [identical to above except for the addition of 1 PhosSTOP tablet (Sigma-Aldrich, #49068450001) for every 10 ml solution], and pre-cleared for 3 h. The 10% BSA blocking step was omitted. Each lysate was divided in half to allow 450 µl per immunoprecipitation. After a 3-h immunoprecipitation step and the three washes (as above), the resulting proteins were eluted from the beads in 40 µl/tube, for a total of 80 µl per genotype. We removed 4 µl for western blot analysis. The remaining 76 µl were precipitated with the addition of 20 µl TCA, 1 h incubation at 4°C, a 15-min spin at 4°C and a wash with ice-cold acetone before drying the pellet and resuspending in 1× SDS loading buffer. The samples were run 1 cm into an SDS-PAGE gel (separated by empty lanes). The gel was fixed for 1 h at room temperature in 45% water/45% methanol/10% acetic acid and then the roughly 1-cm^2^ section of gel containing the protein mixture was cut out, placed in 1% acetic acid (in water), and brought to the Stanford University Mass Spectrometry core for analysis.

Proteins that came down with the GFP-alone control were excluded from Table 1. Among the proteins listed in Table 1, all but Fest, Cdlc2 and Lut contain predicted RNA-binding domains. Cdlc2 has many hits via yeast two-hybrid (BioGrid; Giot et al., 2003) and was assumed to be too sticky for further study.

### UV cross-linking immunoprecipitation (easyCLIP)

#### Sample preparations

Samples were prepared as follows: 120 pairs of testes per sample were dissected in PBS in a glass cyclops dish in batches of 40 pairs. PBS was removed from the dish, which was then put on ice inside a Stratalinker 1800 for UV crosslinking (0.4 J/cm^2^). The testes were then transferred to a 1.7 ml tube with PBS using forceps. PBS was removed and the sample snap-frozen in liquid nitrogen. Samples were kept at −80°C until the next step.

The immunoprecipitation was carried out as described above, without the addition of RNAse or RNAse inhibitor, up to but not including the post-immunoprecipitation washes. The easyCLIP protocol was followed as described by Porter and colleagues (Porter et al., 2021), starting on step 6 of the Basic Protocol 1.

#### easyCLIP pilot

After immunoprecipitation, a strong RNase I digestion was performed (0.1 U/μl, 10 min, 30°C, Thermo Fisher Scientific, #AM2294), followed by on-bead ligation of fluorescent labeled 5′ adapters (L5.27). After an SDS-PAGE gel and transfer to a nitrocellulose membrane, fluorescently labeled crosslinked RNA was imaged on an Odyssey CLx scanner. Western blotting was performed on the membrane following the protocol above.

The pilot CLIP experiment was performed for all four proteins – Syp, Rbp4, Fest and Lut – to assay for RNA-binding activity and to see whether any would be suitable for follow-up to generate sequencing libraries. Fig. S3A shows an image of eYFP-Syp-RNA crosslinked membrane, with signal from western blotting (anti-GFP) on the left, and signal from fluorescent L5 adapter on the right. An orange asterisk marks where there is RNA signal associated with crosslinked eYFP-Syp-RNA. Rbp4-eYFP, V5-Lut and Fest were not included in the follow-up library round of CLIP due to competing nearby bands (Rbp4), weak signal (Lut) or no signal (Fest).

#### easyCLIP library prep

After immunoprecipitation, a light RNase I digestion was performed (0.02 U/μl, 3 min, 30°C), followed by on-bead ligation of 3′ adapters (L3). The two eYFP-Syp replicates were pooled and the two w^1118^ (wt) replicates were pooled, with the genotypes kept separate, before ligating fluorescently labeled 5′ adapters (L5). After an SDS-PAGE gel and transfer to a nitrocellulose membrane, fluorescently labeled crosslinked RNA was imaged on an Odyssey CLx scanner. Fig. S3B shows an image of a nitrocellulose membrane with eYFP-Syp-RNA crosslinked on the left and w^1118^ on the right. The membrane was then cut (rectangles indicated in white on Fig. S3B), and proteinase K (Thermo Fisher Scientific, #AM2546) was used to extract RNA. The RNA was purified using oligonucleotide(dT) beads (Thermo Fisher Scientific, #61002) to capture the poly(A) sequence on the 3′ adapter, eluted, reverse transcribed and input directly into PCR for library preparation. The easyCLIP protocol from Porter et al. (2021) was followed with two modifications: (1) we included NP-40 (0.05%) erroneously omitted from the NT2 buffer in the original protocol and (2) two rounds of PCR were carried out instead of just one round. The first round of PCR was performed with the primers TCGTCGGCAGCGTCAGATGTGTATAAGAGACAG and GTTCAGACGTGTGCTCTTCCGATCT for 5 s at 98°C, then five cycles of: 5 s at 98°C, 5 s at 58°C, 45 s at 72°C, then nine cycles of: 5 s at 98°C, 5 s at 60°C, 45 s at 72°C. The PCR product was then run on a 4% E-gel (Thermo Fisher Scientific, #G401004) to purify products with mappable inserts, followed by four cycles of PCR with full-length index primers NXT_R1_U1/TrSq_R2_U1 for 5 s at 98°C, 1 min at 68°C. The final PCR product was column-purified before sequencing.

#### Primers and adapters

Full-length index primers used were: NXT_R1_U1, AATGATACGGCGACCACCGAGATCTACACTCGTGGAGCGTCGTCGGCAGCGTCAGATGTGTATAAGAGACAG; TrSq_R2_U1, CAAGCAGAAGACGGCATACGAGATCGCTCAGTTCGTGACTGGAGTTCAGACGTGTGCTCTTCCGATCT.

Adapters used in each sample were: eYFP-Syp_rep1: L3.16, L5.1; eYFP-Syp_rep2: L3.24, L5.1; w^1118^_rep1: L3.16, L5.2; w^1118^_rep2: L3.24, L5.2; L3.16:/5Phos/rNrNrNrNrNCATGCAGATCGGAAGAGCACACGTCAAAAAAAAAAAAAAAAAAAAAAAA/3AzideN/; L3.24:/5Phos/rNrNrNrNrNGAGCTAGATCGGAAGAGCACACGTCAAAAAAAAAAAAAAAAAAAAAAAA/3AzideN/; L5.1: /5Cy55/TCGGCAGCGTCAGATGTGTATAAGAGACAGACTATCrNrNrNrNrNrNrN; L5.2: /5Cy55/TCGGCAGCGTCAGATGTGTATAAGAGACAGCGATGTrNrNrNrNrNrNrN; L5.27: /5AzideN/TCGGCAGCGTCAGATGTGTATAAGAGACAGGTATAGrNrNrNrNrNrNrN. rN denotes a random RNA base; /5Phos/, a 5′ phosphorylation; /3AzideN/, a 3′ terminal azide modification from IDT. L5.27 was labeled with fluorescent dye according to the protocol of Porter et al. (2021). Libraries were sequenced with NOVAseq, PE 150 Illumina Platform.

#### easyCLIP data analysis

After demultiplexing, cutadapt was used to trim the reads and move the UMI to the read name:

cutadapt -ACTGTCTCTTATACACATCTGACGCTGCCGACGANNNNNNNNNNGTGTAGATCTCGGTGGTCGCCGTATCATT -a AGATCGGAAGAGCACACGTCTGAACTCCAGTCACNNNNNNNNNNATCTCGTATGCCGTCTTCTGCTTG --pair-filter=any -u 13 -U 10 -j 1 --rename=“{id}__{r1.cut_prefix}-{r2.cut_prefix}” --minimum-length 14 -o $Output1 -p $Output2 $Input1 $Input2.

Reads were mapped to the *Drosophila melanogaster* genome build dm6 using STAR. Reads that fell within gene regions were counted with STAR using Ensembl annotation BDGP6.84.

star_pass1: STAR --runThreadN 4 --runMode alignReads --genomeDir $star_index --alignSJoverhangMin 10 --alignIntronMax 100000 --alignMatesGapMax 100000 --outFilterMismatchNoverLmax 0.04 --readFilesIn $Input1_trimmed $Input2_trimmed --outFileNamePrefix $pass1_prefix --outSAMtype None.

star_pass2: STAR --runThreadN 4 --runMode alignReads --quantMode GeneCounts --genomeDir $star_index --alignIntronMax 100000 --alignMatesGapMax 100000 --outFilterMismatchNoverLmax 0.04 --sjdbFileChrStartEnd $junction --readFilesIn $Input1_trimmed $Input2_trimmed --outSAMtype BAM SortedByCoordinate --limitBAMsortRAM 10000000000 --outFileNamePrefix $pass2_prefix.

Duplicates were removed using samtools followed by umi_tools: samtools view --bam -h -F 256 -f 64 -F 4 -q 10 -o $Output1 $Input; samtools index $Output1; umi_tools dedup --stdin=$Output1 --stdout=$Output2 --umi-separator=“__” --extract-umi-method=read_id.

Samtools was used to create .bw files for visualization: bamCoverage -b $Input -o $Output --binSize 1 --normalizeUsing CPM -p 4. Reads that fell within gene regions were counted with featureCounts using Ensembl annotation BDGP6.84: featureCounts -t exon -g gene_id -p -a $gtf_file -o $Output $Input.

### RNA-seq library preparation and sequencing

We used 150 pairs of testes from 0-2 day-old male flies per replicate. Library preparation was carried out using the RNeasy Plus Mini Kit from QIAGEN, followed by NEBNext^®^ Ultra™ II Directional RNA Library Prep Kit for Illumina (#E7760S) using the NEBNext^®^ Poly(A) mRNA Magnetic Isolation Module (#E7490S). Sequencing was carried out with NOVAseq, Pe150 Illumina Platform, on two biological replicates.

### RNA-seq data analysis

Adapters and low-quality bases were trimmed with trimGalore: trim_galore --quality 20 --stringency 1 --length 30 --paired_end --clip_R1 3 $Input1 $Input2 --output_dir trim_PE/. Reads were mapped to the *Drosophila melanogaster* genome build dm6 using STAR. Reads that fell within gene regions were counted with STAR using Ensembl annotation BDGP6.84, with star_pass1 and star_pass2 the same as for easyCLIP (above).

RPKM was calculated using as ‘gene size’ the median length of the transcript isoforms of the gene. Samtools was used to create .bw files for visualization (same as for easyCLIP). A list of genes expressed in spermatocytes and spermatids was obtained from single-cell RNA-seq data using the threshold ‘expressed above 0 in at least 10% of [spermatocytes/spermatids]’ (Raz et al., 2023).

### Western blotting

Samples were run on 10% or 4-15% TGX pre-cast gels with ten wells with 50 µl well volume (Bio-Rad, #4561034 and #4561084). Proteins were transferred overnight onto PVDF membrane (wetted briefly with methanol) in methanol-free transfer buffer (25 mM Tris, 192 mM glycine) at 100 mA constant current, at 4°C with stirring. After transfer, the membrane was blocked for at least 1 h in 5% milk in Tris-buffered saline (TBS), then placed in a 4×4 inch resealable plastic zip lock bag, to which 2.5 ml 5% milk/TBS plus primary antibody was added in an even stream across the surface of the membrane. Excess air was released before sealing the bag, and then the antibody solution was massaged back and forth over the membrane for 1 min, and incubated for 1 h at RT. The membrane was rinsed briefly in 5% milk/TBS before a similar incubation step in HRP-conjugated secondary antibody. It was then washed vigorously in TBS on a shaker for 2 h. The membrane was incubated with Western Lightning Plus-ECL detection reagents (Perkin Elmer, #NEL104001EA), sandwiched between layers of Saran Wrap, and exposed to HyBlot CL film (Thomas Scientific, #1141J52). Primary antibodies used were as follows: mouse anti-GFP (Millipore Sigma, 11814460001, 1:2000); rabbit anti-HA (Cell Signaling Technology, 3724S, 1:2000); mouse anti-V5 (Thermo Fisher Scientific, R960-25, 1:1000); guinea pig anti-Syp (Davis lab, McDermott et al., 2014, 1:20,000); mouse anti-Tubulin (Cell Signaling Technology, #3873S, 1:500).

### Immunostaining

For anti-CycB staining, testes were dissected in 1× PBS, fixed in 1 ml ice-cold methanol (5 min), washed in 1 ml ice-cold acetone (2 min) and then washed in 1 ml room-temperature PBS+0.1% Triton (PBSTr). Testes were blocked for 30 min in 1 ml 3% BSA/PBSTr and incubated in anti-CycB (mouse F2F4, Developmental Studies Hybridoma Bank) overnight at 1:50 in 3% BSA/PBSTr at 4°C. After a 1 h wash in 1 ml 3% BSA/PBSTr, testes were incubated in secondary antibody (Alexa Fluor-conjugated donkey anti-mouse 488, Molecular Probes, 1:200) for 2 h in the dark at RT. After two 10-min washes in PBSTr, testes were transferred to a slide, where the liquid was removed and replaced with one drop of DAPI-containing Vectashield (Vector Labs). A 22×40 mm coverslip was placed on top and secured with nail polish. Samples were imaged within a few hours of mounting.

For all other antibodies, testes were dissected in 1× PBS in a 1.7 ml Eppendorf tube, fixed in 1 ml 4% formaldehyde/PBS for 30 min, washed briefly in 1 ml PBSTr, and permeabilized in 1 ml 0.3% sodium deoxycholate/0.3% Triton X-100/PBS for 30 min. After a quick wash in PBSTr, samples were processed through block, primary antibody, wash, secondary antibody, washes and mounting, as described above. Primary antibodies used were: rabbit anti-Syp (Rossi and Desplan, 2020, 1:200), anti-Tj (guinea pig; a gift from D. Godt, University of Toronto, Canada, 1:100), rabbit anti-HA (3724S, Cell Signaling Technology, 1:100) and chicken anti-GFP (Abcam, #13970, 1:10,000). Alexa Fluor-conjugated secondary antibodies – donkey anti-rabbit 488, anti-guinea pig 549, anti-chicken 488 and anti-rabbit 568 – from Molecular Probes were used at 1:200.

### Single-molecule FISH

Testes were dissected from 1-2 day old flies in 1× PBS, fixed in 5% formaldehyde for 30 min, then permeabilized by incubation in PBS with 0.3% Triton X-100 and 0.6% sodium deoxycholate for 30 min at RT. After permeabilization, we followed the conventional smFISH protocol in *Drosophila* (Trcek et al., 2017) while using the primary/secondary probe strategy from the inexpensive version (Tsanov et al., 2016; Gallicchio et al., 2021). Samples were mounted using 10 µl of ProLong™ Diamond Antifade Mountant with DAPI (Thermo Fisher Scientific). Primary probes against the *cycB* and *loopin-1* RNAs (listed in Tables S1 and S2, respectively) were designed using the Biosearch Technologies Stellaris probe Designer (version 4.2). The Flap sequence TTACACTCGGACCTCGTCGACATGCATT was added to the 5′ end of each probe. A secondary probe complementary to the Flap sequence was tagged with fluorophore CAL Fluor Red 610.

Imaging of smFISH samples was performed using a Leica SP8 Inverted Tandem Head confocal microscope and LAS X v3.5.7.23225 software (Cell Science Imaging Facility, Stanford University). Images were all taken at 40× magnification using the built-in tiling function to reconstruct the entire testis. The quantification of smFISH signal in Figs 5 and 9 was performed on three testes per genotype, then averaged to give the values shown. An *n* of three testes for imaging was chosen because of the reliability of the technique and limited time on a shared resource. Relative fluorescence intensity was calculated for each testis by using FIJI to measure mean gray value (MGV) in squares of mid-stage and late-stage spermatocyte cytoplasm (5+ per testis) as well as squares of background staining (3+ per testis) from early spermatocytes (which have not yet turned on *cycB* from the spermatocyte promoter), then subtracting the average MGV of background from the average MGV of mid/late spermatocytes.

### Microscopy

Images from immunostaining and phase-Hoechst staining were captured by a Photometrics CoolSNAP CCD camera connected to a Zeiss Axioskop microscope, with fluorescence illumination provided by an X-Cite 120 excitation light source. Phase imaging was conducted on the above setup (Fig. 4A-D) or with a Spot RT3 CCD camera affixed to a Zeiss Axioskop microscope (Fig. 4G-I, Fig. 5D-F). For Fig. 5D-F, overlapping photos were taken at 20× and stitched together in Photoshop Elements. For scoring meiotic entry and exit (Fig. 4), nuclei were not considered round enough to count as ‘meiotic entry’ if they were olive or teardrop shaped, or if they had a lot of wobble when the focus shifted along the *z*-axis. Cells were not scored as round spermatids if the mitochondrial derivative was smudgy or lacked a smooth circumference.

## Supplementary Material

10.1242/develop.201709_sup1 Supplementary information
