# Supplementary material for: Cell-type-specific interacting proteins collaborate to regulate the timing of Cyclin B protein expression in male meiotic prophase
Source: Development. 2023 Nov 27;150(22):dev201709. doi: 10.1242/dev.201709 (PMC10730016; doi:10.1242/dev.201709)
Supplement: Supplementary information [file develop-150-201709-s1.pdf]

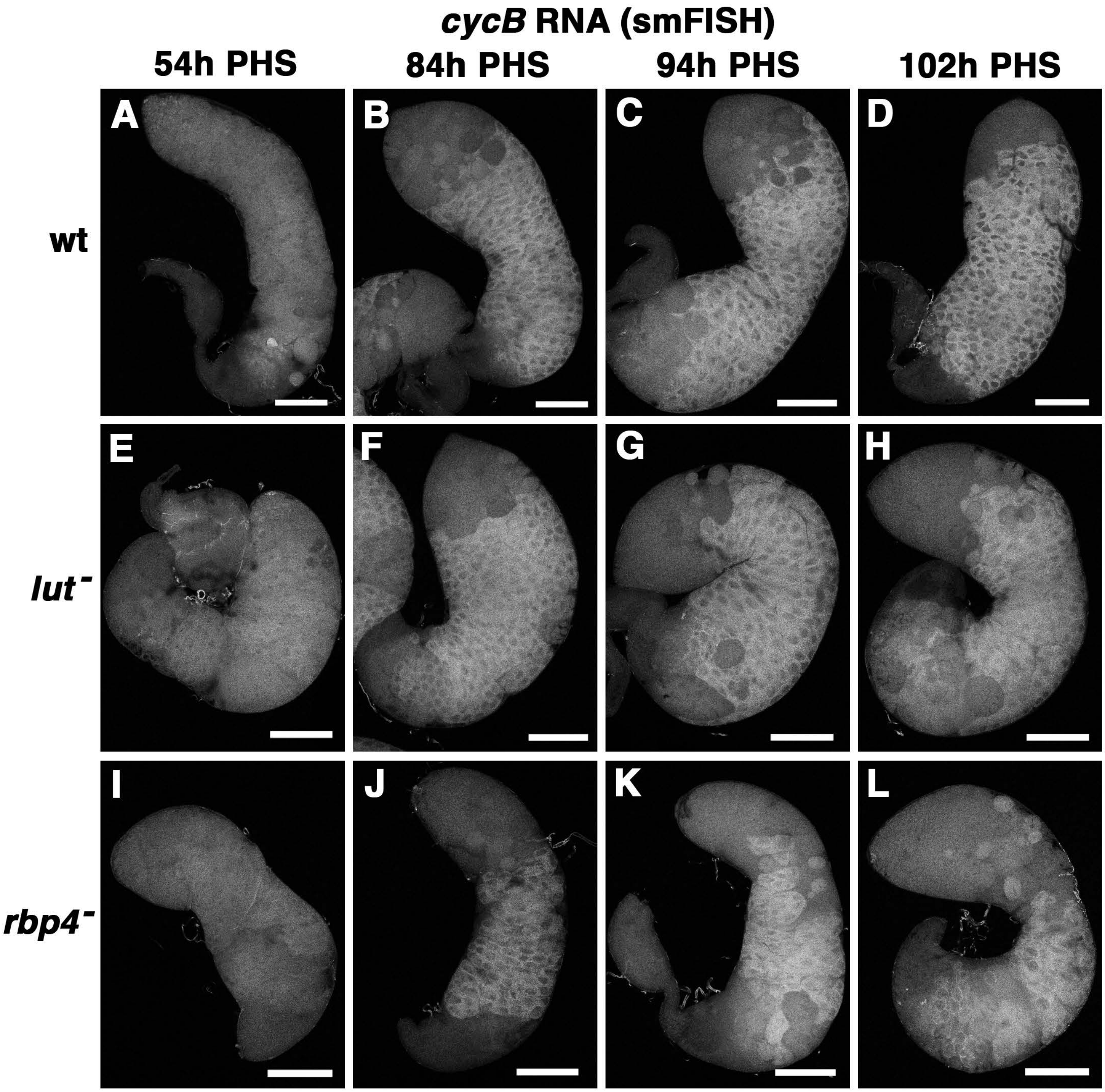

Fig. S1. smFISH with *cycB* probes on (A-D) wt, (E-H) *lut*, and (I-L) *rbp4* mutants at 54h, 84h, 94h, and 102h PHS. Scale bars: 100 µm.

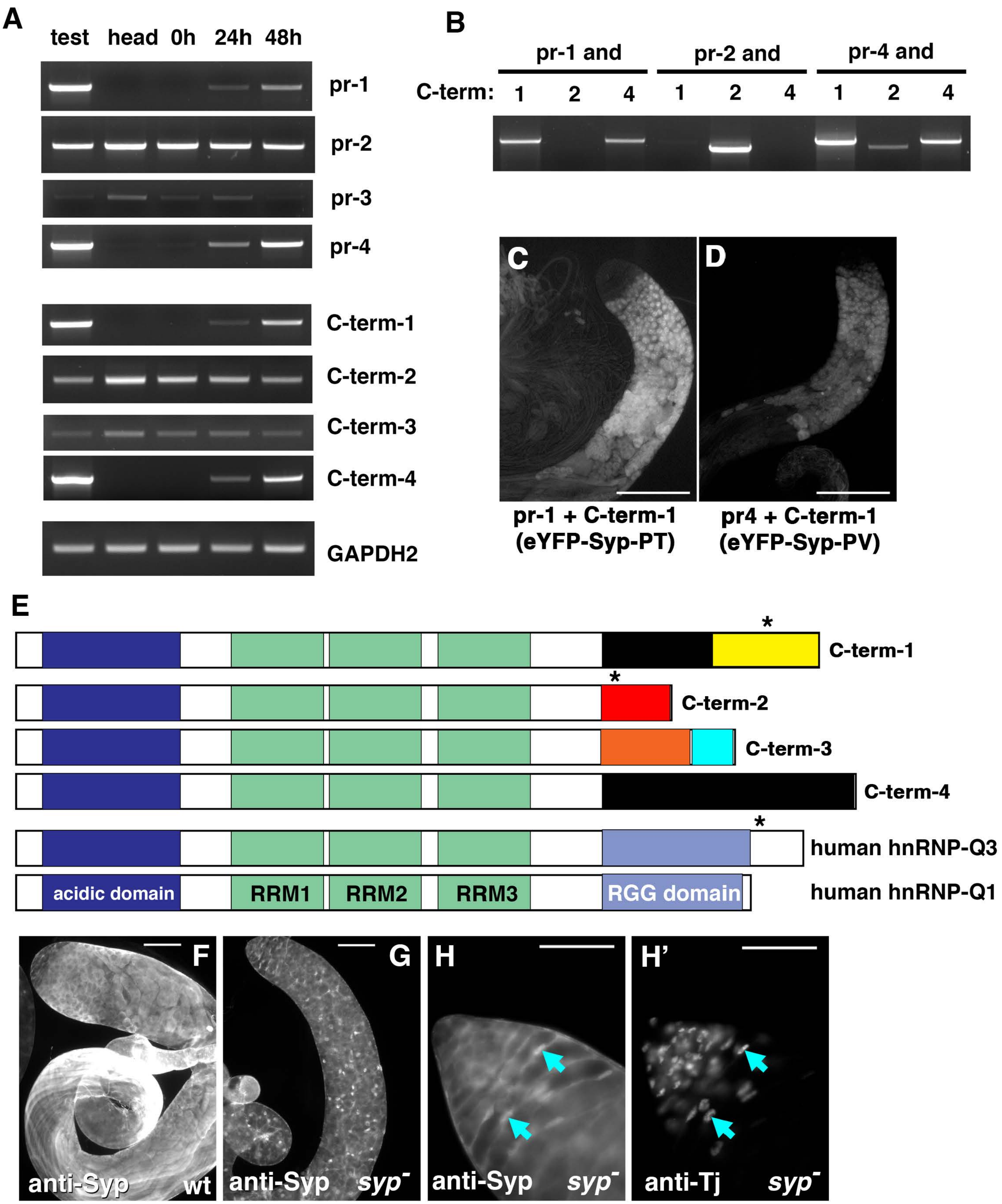

Development • Supplementary information

Fig. S2. (A) RT-PCR of the four *syp* promoters (rows 1-4), the *syp* C-terminal variants (rows 5- 8), and *GAPDH2* (last row) from cDNA from testis, head, and time-course testes (0h [*bam*], 24h, and 48h PHS). (B) RT-PCR from testis cDNAs for several pairwise combinations of promoters and C-termini, as indicated. (C,D) live fluorescence imaging of (C) eYFP-Syp-PT (promoter 1 + C-term-1) and (D) eYFP-Syp-PV (promoter 4 + C-term-1). Both reporter proteins localized to the nucleus in early spermatocytes (consistent with the NLS encoded in C-term-1) but largely shuttled to the cytoplasm in later spermatocytes. Scale bar: 200 µm. (E) Diagram of predicted Syp proteins, with two isoforms of human HNRNPQ at bottom. Black asterisks indicate nuclear localization signals (NLSes). The acidic domain is enriched for aspartic acid and glutamic acid residues; the RRM domains are classic RNA-binding domains; and the RGG domain in human HNRNPQ is enriched in arginine-glycine-glycine repeats. (F,G) anti-Syp (rabbit) on (F) wild-type and (G) *syp* mutant testes. Scale bar: 100 µm. (H,H') High-magnification view of the testis apical tip from *syp* mutant male stained with (H) rabbit anti-Syp and (H') anti-Tj. Arrows: somatic nuclei expressing both proteins. Scale bar: 50 µm (H,H').

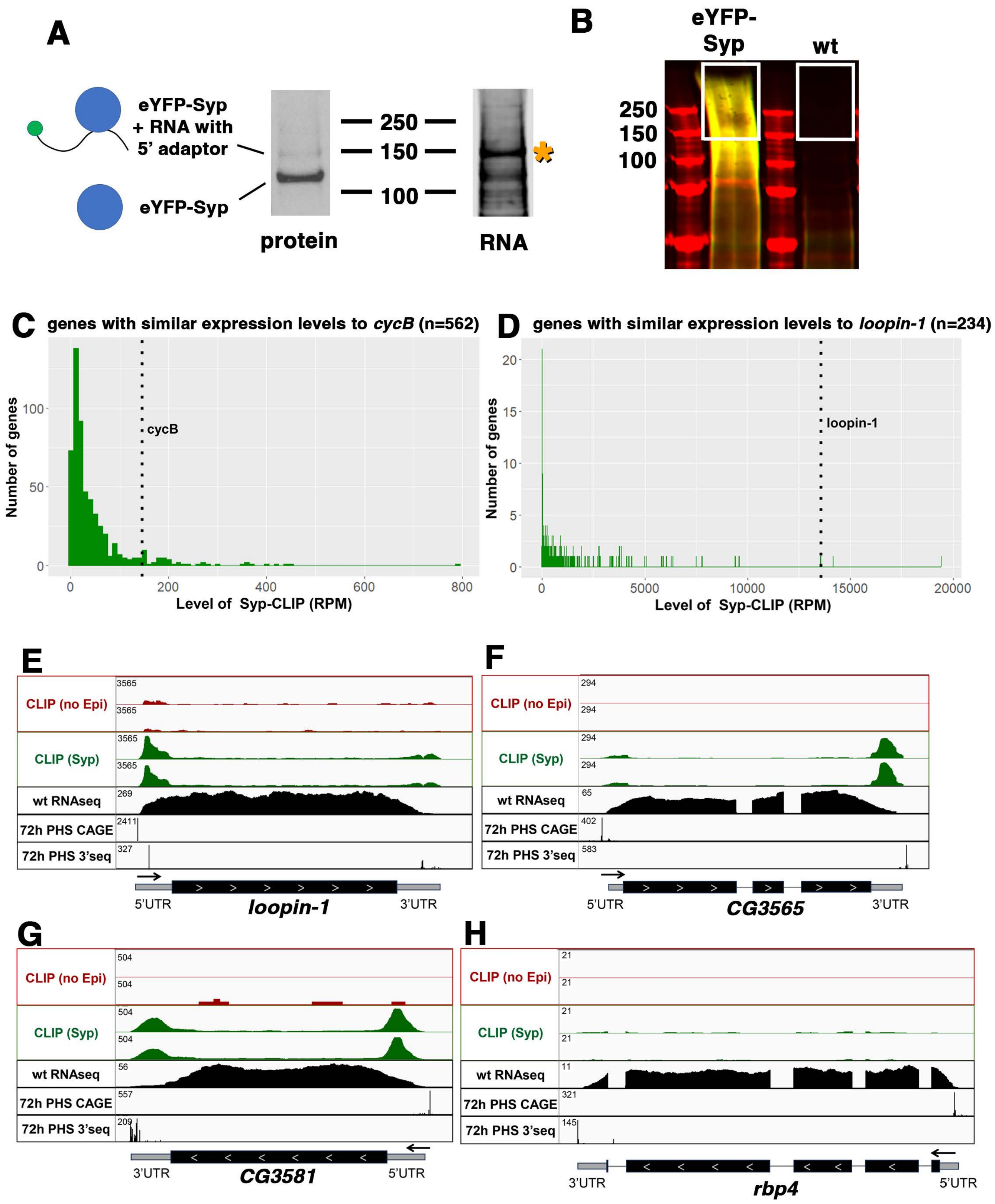

Development • Supplementary information

Fig. S3. Supplemental CLIP data and methodology. (A) Images of membrane from CLIP pilot experiment, showing (left) eYFP-Syp protein (western blot) and (right) RNA bound to eYFP-Syp (orange asterisk). eYFP-Syp alone migrated at ~135 kD; eYFP-Syp bound to RNAs with a 5' adapter attached migrated at ~150 kD. (B) Image of membrane from easyCLIP experiment showing RNAs recovered with immunoprecipitated eYFP-Syp (left lane). The portions of the membrane marked with white boxes were cut out to make libraries for both eYFP-Syp and w^{1118} (no epitope) control samples (see Methods). (C) Histogram of 562 spermatocyte-expressed genes with similar expression levels to *cycB* (*cycB* expression level -/+ 5 RPKM), distributed along the x axis by increasing abundance of Syp CLIP level (RPM). Level of Syp-CLIP at *cycB* is marked by a black vertical dotted line. (D) Histogram of 234 spermatocyte-expressed genes with similar expression levels to *loopin-1* (*loopin-1* expression -/+ 700 RPKM), distributed along the x axis by increasing abundance of Syp CLIP level (RPM). Level of Syp-CLIP at loopin-1 is marked by a black vertical dotted line. (E-H) IGVs of (E) *loopin-1* (F) *CG3565* (G) *CG3581* (H) *rbp4*. IGV tracks are the same as in Figure 5Q.

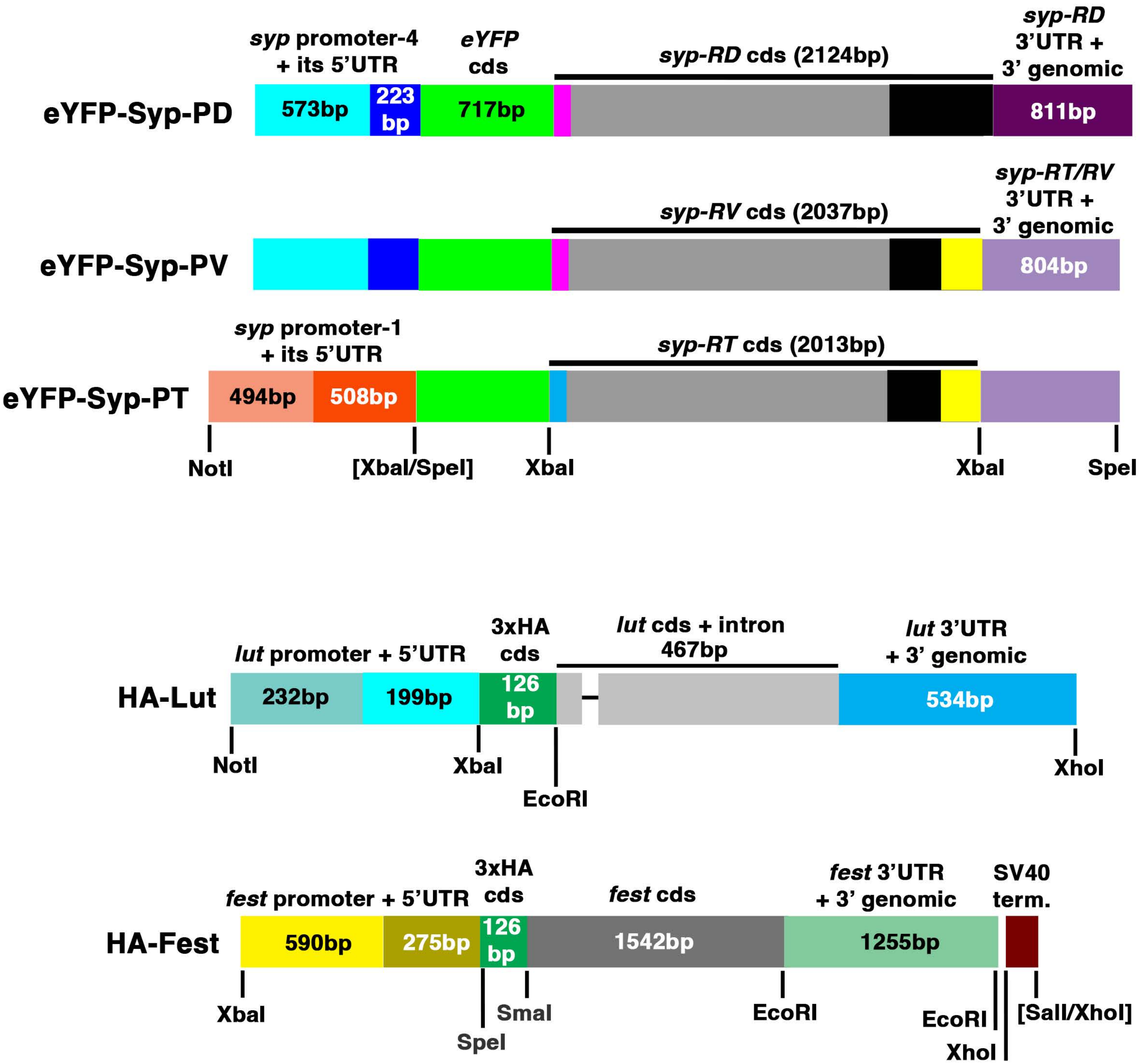

Fig. S4. Diagram of new transgenes described in Materials and Methods. Not shown: V5- Lut, which is similar to HA-Lut but with a V5 coding sequence (42bp) in place of 3xHA.

Development • Supplementary information

Table S1. *cycB* primers for smFISH.

TTACACTCGGACCTCGTCGACATGCATTgattctgcaaatcgcccaag
TTACACTCGGACCTCGTCGACATGCATTctgcgatgggacgacttatg
TTACACTCGGACCTCGTCGACATGCATTttgagatccttggagtcttt
TTACACTCGGACCTCGTCGACATGCATTcgaacgcaaaaacgccgaca
TTACACTCGGACCTCGTCGACATGCATTtcttggtcggaacgcgattg
TTACACTCGGACCTCGTCGACATGCATTactttagtgggttctacagt
TTACACTCGGACCTCGTCGACATGCATTctcggaagaactggacttga
TTACACTCGGACCTCGTCGACATGCATTtttaaggtgggctcgttcac
TTACACTCGGACCTCGTCGACATGCATTagcgacttcttcgacagatt
TTACACTCGGACCTCGTCGACATGCATTcaaagcggcacgcagtttgg
TTACACTCGGACCTCGTCGACATGCATTaaactcccatcacgggtttg
TTACACTCGGACCTCGTCGACATGCATTtactggttcccgtcgaattc
TTACACTCGGACCTCGTCGACATGCATTtctgcctctttgcgggaaac
TTACACTCGGACCTCGTCGACATGCATTttcttggtttctggcagttc
TTACACTCGGACCTCGTCGACATGCATTctttttcacttccagtgagt
TTACACTCGGACCTCGTCGACATGCATTtaataaggggcatcctggtc
TTACACTCGGACCTCGTCGACATGCATTcgtagtgactgcactgttgc
TTACACTCGGACCTCGTCGACATGCATTtggtgggcatcgtagatgtg
TTACACTCGGACCTCGTCGACATGCATTcgcttgctggaaagggacat
TTACACTCGGACCTCGTCGACATGCATTtcaatgtcctcgattccagc
TTACACTCGGACCTCGTCGACATGCATTcaggttctccttgtcattgg
TTACACTCGGACCTCGTCGACATGCATTcgtttacatattcggagacc
TTACACTCGGACCTCGTCGACATGCATTtccacctgatacaagtagtc
TTACACTCGGACCTCGTCGACATGCATTcaggtgatccttgtgaatgg
TTACACTCGGACCTCGTCGACATGCATTcaatcgatcagcacggctcg
TTACACTCGGACCTCGTCGACATGCATTactgcaggtggacttcgttg
TTACACTCGGACCTCGTCGACATGCATTaaggtctctgcagccagatg
TTACACTCGGACCTCGTCGACATGCATTgtagcgatcaatgatagcca
TTACACTCGGACCTCGTCGACATGCATTattgcaagtacgtgcgtttg
TTACACTCGGACCTCGTCGACATGCATTgctatgaagagtgctgtcac
TTACACTCGGACCTCGTCGACATGCATTggaacagctcctcgtacttg
TTACACTCGGACCTCGTCGACATGCATTgacgaaatctccgattgccg
TTACACTCGGACCTCGTCGACATGCATTtgtaggtgtcgtccgtgatg
TTACACTCGGACCTCGTCGACATGCATTttgaagatttgcagctccat
TTACACTCGGACCTCGTCGACATGCATTcgcgacagattacagtcgat
TTACACTCGGACCTCGTCGACATGCATTaaggaagtgaatcggcagcg
TTACACTCGGACCTCGTCGACATGCATTgtacttggacatcgtatggt
TTACACTCGGACCTCGTCGACATGCATTccacggaagctaactcgatg
TTACACTCGGACCTCGTCGACATGCATTctgtaagtggccatttcgta
TTACACTCGGACCTCGTCGACATGCATTcgacaggaacagtgaggcag

Development • Supplementary information

TTACACTCGGACCTCGTCGACATGCATTtggtttccattgagcaagtg
TTACACTCGGACCTCGTCGACATGCATTacggtcgttgaatcctgtac
TTACACTCGGACCTCGTCGACATGCATTgagtatcgcgagtagaaggt
TTACACTCGGACCTCGTCGACATGCATTcgggtaatcggacgcaagtg
TTACACTCGGACCTCGTCGACATGCATTtggtacttgttgtagatggc
TTACACTCGGACCTCGTCGACATGCATTcgatcttctggaacttgctg
TTACACTCGGACCTCGTCGACATGCATTcacaatcgagtccatcagcg

Development • Supplementary information

Table S2. ***loopin-1*** **primers for smFISH**

TTACACTCGGACCTCGTCGACATGCATTatctgtgacccgtacgaaat
TTACACTCGGACCTCGTCGACATGCATTacgcgcgtatttttttcacaa
TTACACTCGGACCTCGTCGACATGCATTaatacgtttgttggtcacct
TTACACTCGGACCTCGTCGACATGCATTcgcaccaaagacatgttctt
TTACACTCGGACCTCGTCGACATGCATTtgtccagaacgattttggcg
TTACACTCGGACCTCGTCGACATGCATTgcgatatgacggtatcacag
TTACACTCGGACCTCGTCGACATGCATTtcttgcaggatgtcgcatag
TTACACTCGGACCTCGTCGACATGCATTccttggtataaacaccaacc
TTACACTCGGACCTCGTCGACATGCATTtggttttcgatggcttatcg
TTACACTCGGACCTCGTCGACATGCATTatccaaggtcactgcatttg
TTACACTCGGACCTCGTCGACATGCATTtcacggatcagggtcaacag
TTACACTCGGACCTCGTCGACATGCATTgaagccactgaagagcagac
TTACACTCGGACCTCGTCGACATGCATTtgtttgccaacaccgacaac
TTACACTCGGACCTCGTCGACATGCATTgttcctcgttttcattgtag
TTACACTCGGACCTCGTCGACATGCATTatagtccatggaatccacat
TTACACTCGGACCTCGTCGACATGCATTgttggcattataacgccata
TTACACTCGGACCTCGTCGACATGCATTtctgaatgcggttcttcttc
TTACACTCGGACCTCGTCGACATGCATTtgccatacatatcgagcttg
TTACACTCGGACCTCGTCGACATGCATTggacaaacgtctggccaaat
TTACACTCGGACCTCGTCGACATGCATTaatatcgagggcgtcatcat
TTACACTCGGACCTCGTCGACATGCATTttgaggttcatatcctcgat
TTACACTCGGACCTCGTCGACATGCATTccttggcgatcattaggaag
TTACACTCGGACCTCGTCGACATGCATTaatagaggtggttcgcacga
TTACACTCGGACCTCGTCGACATGCATTaccactgtggaaggtcagac
TTACACTCGGACCTCGTCGACATGCATTaacacccacacaaacagcag
TTACACTCGGACCTCGTCGACATGCATTatgttcagcgggagcgaaag
TTACACTCGGACCTCGTCGACATGCATTcttgtccacattcttgatgc
TTACACTCGGACCTCGTCGACATGCATTatatcgatgaccagtttggg
TTACACTCGGACCTCGTCGACATGCATTgccaacggcatagttaacac
TTACACTCGGACCTCGTCGACATGCATTaagttctgccacacagactt
TTACACTCGGACCTCGTCGACATGCATTacttgaacagggggaaacgc
TTACACTCGGACCTCGTCGACATGCATTcttgttcacgatctgtttgt
TTACACTCGGACCTCGTCGACATGCATTgttgcacagatcataggtga
TTACACTCGGACCTCGTCGACATGCATTaaccagggtgtgaaggattg
TTACACTCGGACCTCGTCGACATGCATTggttgattgtggtggtcatg
TTACACTCGGACCTCGTCGACATGCATTatcgagtccttgagtaggta
TTACACTCGGACCTCGTCGACATGCATTgaactggataacagtgcgcg
TTACACTCGGACCTCGTCGACATGCATTaggaaggtgtgtctacatgg
TTACACTCGGACCTCGTCGACATGCATTaatacatgggccaacatgga
TTACACTCGGACCTCGTCGACATGCATTctgtgcaaacactgtaccaa
TTACACTCGGACCTCGTCGACATGCATTtctccagaccagaatgtttt
TTACACTCGGACCTCGTCGACATGCATTgtgaacctttacgaggatgg
TTACACTCGGACCTCGTCGACATGCATTctcaagttaccacatttgct
TTACACTCGGACCTCGTCGACATGCATTttgattcgataggtcggttc
TTACACTCGGACCTCGTCGACATGCATTggccatatttatacgattgg
TTACACTCGGACCTCGTCGACATGCATTtgccatcaatcgagttggaa
TTACACTCGGACCTCGTCGACATGCATTtcaaagccaggaaaatccgc
TTACACTCGGACCTCGTCGACATGCATTtcactccagccaaaaggtaa

Development • Supplementary information
