# Supplementary material for: Cell-type-specific interacting proteins collaborate to regulate the timing of Cyclin B protein expression in male meiotic prophase
Source: Development. 2023 Nov 27;150(22):dev201709. doi: 10.1242/dev.201709 (PMC10730016; doi:10.1242/dev.201709)
Supplement: Supplementary file 1 [file develop-150-201709_review_history.pdf]

Cell-type-specific interacting proteins collaborate to regulate the timing of Cyclin B protein expression in male meiotic prophase

Catherine C Baker, Lorenzo Gallicchio, Neuza R Matias, Douglas F Porter, Lucineh Parsanian, Emily Taing, Cheuk H Tam and Margaret T Fuller
DOI: 10.1242/dev.201709

Editor: Swathi Arur

Review timeline

Original submission:	20 February 2023
Editorial decision:	23 March 2023
First revision received:	18 September 2023
Accepted:	10 October 2023

Original submission

First decision letter

MS ID#: DEVELOP/2023/201709

MS TITLE: A cell-type-specific multi-protein complex regulates expression of Cyclin B protein in Drosophila male meiotic prophase

AUTHORS: Catherine C Baker, Lorenzo Gallicchio, Lucineh Parsanian, Emily Taing, Cheuk H Tam, and Margaret T Fuller

I have now received all the referees' reports on the above manuscript, and have reached a decision. The referees' comments are appended below, or you can access them online: please go to BenchPress and click on the 'Manuscripts with Decisions' queue in the Author Area.

As you will see, the referees express interest in your work but also provide significant recommendations that greatly enhance the rigor and analysis of the study. If you are able to revise the manuscript along the lines suggested, which may involve further experiments, I will be happy receive a revised version of the manuscript. Your revised paper will be re-reviewed by one or more of the original referees.

Please attend to all of the reviewers' comments and ensure that you clearly highlight all changes made in the revised manuscript. Please avoid using 'Tracked changes' in Word files as these are lost in PDF conversion. I should be grateful if you would also provide a point-by-point response detailing how you have dealt with the points raised by the reviewers in the 'Response to Reviewers' box. If you do not agree with any of their criticisms or suggestions please explain clearly why this is so.

Reviewer 1

Advance summary and potential significance to field

Baker et al. characterize the spermatogenesis phenotype of mutants that they generated in Drosophila lutin and in Syncrip. They show that each mutant causes a change in the timing of meiosis exit. They present data that mutation of lut affects the timing of translation of CycB mRNA in spermatocytes, and that Syp has spermatocyte-specific isoforms that stabilize CycB mRNA. They

© 2023. Published by The Company of Biologists under the terms of the Creative Commons Attribution License (https://creativecommons.org/licenses/by/4.0/).

carry out immunoprecipitation experiments between Lut or Syp and Rbp4 and Fest, which were previously shown to regulate CycB translation and to interact. They report that like Rbp4, Syp binds the 3' UTR of CycB RNA, that Lut and Fest co-precipitate in an RNA-independent manner. They show that in the presence of Fest, Lut can co-precipitate Rbp4, consistent with the three proteins forming a complex. Syp also co-precipitates with Fest, and to thus interact with Lut and Rbp4. The authors posit that the proteins form a complex associated with the 3'UTR of CycB mRNA that controls the translation and/or stability of that RNA. They suggest three models that are consistent with the co-IP data. They posit that the complex controls the timing of meiosis. Lut mutants accelerate CycB appearance and meiosis exit by several hours, whereas syp mutant arrests meiosis.

The work in this paper is thorough and carefully done. The results are new believable, and interesting. But three major items need to be addressed before the manuscript would be suitable for publication, and some clarifications to the writing or figures are needed to make it easier to comprehend.

Comments for the author

Major points to address

1. There is only a small difference in timing (~8%) between meiosis exit in mutants relative to controls or each other. Can the authors provide information to assure the reader that the synchronization has the precision to detect such a small timing difference? Are the strains sufficiently identical to remove concern about genetic background effects on the timing? Related, the inference of timing of CycB expression in Figure 1 G-I would be more convincing if the figure showed the cell-types where expression is detected (i.e. by morphology) or if the location/spacing of CycB expression was quantified.
2. The co-immunoprecipitations are done on extracts. It seems possible that the proteins interact in vivo within different complexes that are incorrectly amalgamated in Fig. 2c, the title of the paper, and elsewhere. Perhaps some Rbp4 interacts with the 3' UTR of CycB mRNA, and other Rbp4 is in a complex with Lut.
Can the authors provide evidence that the proteins always co-localize in the cell with each other and with CycB mRNA?
3. The authors need to establish that Cyclin B expression is sufficient to drive the meiotic program – whether through citations or experiments.

Minor points to address

1. Aspects of the data/writing that could be better described/explained
a. The band in the negative control in Figure 2B seems problematic for their interpretation.
b. Figure 3 A-D – there is an apparent expansion of CycB staining in spermatogonia at the later time points
c. Line 189-190 – more explanation of what this implies, full name references
d. Fig 4E-F "at least one cyst" is a reasonable way to quantify this data but please, at least qualitatively, describe how similarly the cysts behaved – they would be expected to be synchronized.
e. More explanation/justification is needed tfor why expression of Syp in heads in the syp mutant is described as surprising.

2. Suggestions for tightening up the writing:
a. Refer to the timings in a consistent way. "PHS" is a great way to indicate that you are using and referring to the bam-HS system, but you only use this on occasion. I would be more consistent i.e. in Figure 1D,E, Figure 3, 6 etc.
b. Line 141 – The topic sentence of this paragraph could better distinguish the experiments in this paragraph from those directly above. i.e. you are now using the hs-Bam system to narrow in on the timing of the interactions
c. Line 142 – Suggest mentioning/contrasting the CycB RNA expression pattern here.
d. The genes in the proposed seemed to arise out of the blue – please describe better why they were tested/studied

© 2023. Published by The Company of Biologists under the terms of the Creative Commons Attribution License (https://creativecommons.org/licenses/by/4.0/).

e. Paragraph 189-201 vs 202-207 – Slightly different language is used to describe the same thing. If you mean that they are the same, you can write this more concisely. If you mean that they are different, please clarify how.
f. The study of the genes in this paper seems to come out of left field. I would appreciate more explanation of how you arrived at these genes.

3. Figure 2F : “10% input” across the top left vs bottom right – I’m assuming the top left is a typo?
4. The lut CRISPR mutant has not been demonstrated to be a null. You can refer to it as a “likely null” or give it an allele number.

5. I don’t follow what the arrows are supposed to show in Fig 7A’-D’

6. The sample shown in Figure 9K looks poorly fixed, which can lead to higher background staining. I’m concerned about the quantification of expression levels from samples such as this.

7. In Fig8A it looks like syp is upregulated in rbp4-/-. Could this indicate competitive binding with Fest?

<u>Reviewer 2</u>

Advance summary and potential significance to field

This manuscript describes the characterization of several RNA binding proteins that physically interact to control the translation of cyclin B within the developing germline of Drosophila males. Cyclin B is translationally repressed in immature Drosophila spermatocytes and previous work from the lab showed that Rbp4 and its partner Fest can bind to elements within the 3’UTR of cyclin B mRNA. The work presented here extends these earlier findings and shows that another spermatocyte-specific protein, Lut, also participates in the repression of cyclin B during an 8 hour window of time, just before spermatocytes are mature. In addition, spermatocyte-specific isoforms of Syncrip regulate the normal expression of cyclin B and entry into meiosis. Lastly, the authors provide evidence that Lut and Syp interact with Fest in an RNA-independent manner.

This study will be of general interest to the readership of Development. Understanding how different RNA binding proteins coordinate to regulate the stage and tissue-specific translation of mRNAs is of great interest to the field. This study provides further insights into the regulated translation of cyclin B mRNA as germ cells transition into meiosis. However, several points should be addressed prior to publication.

Comments for the author

The study has many moving parts with 10 figures and 2 supplemental figures. The transition from the Lut data (Figs 1-4) to the Syp data (Figs 5-9) should be improved. Simplifying and consolidating the figures may result in a clearer and more cohesive presentation of the overall story.

One of the primary weaknesses of the paper is that the authors do not provide strong evidence that these factors directly bind to the 3’UTR of cyclin B in vivo. Beyond the biotinylated pulldowns shown for Syp, the authors should perform RNA-immunoprecipitation for Rbp4, Fest, Lut, and Syp in the presence of crosslinking agents to show that these factors indeed interact with endogenous cyclin B mRNA.

Additional structure/function analysis would bolster the claim that Rbp4, Fest, Lut and Syp form a complex in which Fest acts as a scaffold. Specific mutations in Fest that disrupt its interactions with other complex members should be made and tested for functional significance.

Does the HA-lut transgene first presented in Figure 1 rescue the lut mutant phenotype? If so, these data should be presented. Are lut males completely sterile? A brief description of the overall fertility of these mutants should be included in the paragraph describing the generation of the null mutant (lines 119-126).

© 2023. Published by The Company of Biologists under the terms of the Creative Commons Attribution License (https://creativecommons.org/licenses/by/4.0/).

In Figure 2A, treatment with RNAse appears to increase the interaction between Lut and Fest. Do the authors have an explanation for this?

The IP presented in Figure 2B is relatively weak (IP shows lower levels of HA-Fest relative to 10% input).

The authors could consider moving this panel into supplemental data or removing it altogether.

Further biochemical experiments are needed to support the model presented in Fig 2C. It remains unclear whether all three proteins co-assemble into a single complex or whether sub-complexes form.

The quality of the image presented in Fig 2E does not match other images throughout the paper. The authors should provide another image that convincingly shows Cyclin B expression at 104 hrs after hs-bam induction.

In Fig 2F- the “10% input” at the top of the panel may be misplaced.

For Figure 3- The authors should provide data describing the levels of cyclin B mRNA in the controls and mutants at all the timepoints. Some of the control data are presented in figure 6. Knowing the RNA levels in the mutants in Fig 3 would strengthen the interpretation of these data.

The authors state, “In lut mutants, however, round spermatids typically had four nuclei and one large mitochondrial derivative per cell (Fig. 4H), suggesting complete failure of cytokinesis after meiosis I and II. In contrast, the rbp4 mutant showed evidence of much milder cytokinesis defects (Fig. 4I, arrows).” Given that cyclin B protein expression accumulates at earlier times in the rbp4 mutant relative to lut, can the authors provide a further explanation of how the lut phenotype relates to misexpression of Cyclin B protein?

In panels Fig 8B and Fig 8D, the levels of Syp appear to increase in the absence of rbp4 and fest respectively. Do the authors think each of these proteins influence the levels of one another? Does Lut influence the levels and/or interactions of Fest and Syp?

Data presented in figure 9 suggest that Rbp4, Lut, and Syp are needed for the stability of cyclin B mRNA.

Why these levels appear to increase in the lut syp double mutants (Fig 9K) relative to the single mutants is unclear. Attempting to quantify mRNA levels using mean fluorescence is problematic without internal controls. Additional methods should be used to make this point because the presented images do not appear to match the quantification provided in Fig 9M. Statistics are needed in Fig 9M as well.

The models presented in Figure 10 suggest that Lut represses cyclin B translation independent of rbp4. Do the authors mean to suggest that the physical interactions between rbp4 and lut (bridged by Fest) described in Fig 2 are not functionally significant? Further discussion along these lines is warranted.

First revision

Author response to reviewers' comments

We thank the reviewers for their useful suggestions. In response we have made major updates to the paper, adding new results. These include easyCLIP data showing direct binding of Syp to the *cycB* RNA *in vivo*; smFISH against *cycB* RNA to match all of the timepoints and genotypes used for anti-CycB immunostaining in Figure 3; smFISH against *cycB* for Figure 2 to match the 72h and 104h

© 2023. Published by The Company of Biologists under the terms of the Creative Commons Attribution License (https://creativecommons.org/licenses/by/4.0/).

PHS anti-CycB immunostaining; and a repeat of smFISH (*cycB* and *loopin-1*) for Figure 9. In addition, we made substantial revisions to the text to address reviewers' concerns. Major changes to the text of the manuscript include detailed explanations of how we came to be interested in *lut* and *syp*; additional details in the Materials and Methods about both the hs-Bam time- course and the ability of the HA-Lut transgene to rescue the *lut* mutant; and removal of speculative models from figures and text showing Lut and Fest recruited to the *cycB* RNA via Rbp4/Syp. In addition, in the absence of data showing co-sedimentation through a velocity gradient or co-migration through a sizing column, we have removed the term "complex" from the title and throughout the manuscript. Although Lut, Fest, Rbp4, and Syp proteins clearly interact, we cannot prove that they form a monolithic unified complex at all stages of their action.

See below for point-by-point responses (blue text) to reviewers' comments and questions (black text). In the manuscript itself, updates / changes to the text requested by the reviewers are in red; sections re-written for clarity or moved to methods are in blue.

Rev1

The work in this paper is thorough and carefully done. The results are new, believable, and interesting. But three major items need to be addressed before the manuscript would be suitable for publication, and some clarifications to the writing or figures are needed to make it easier to comprehend.

Reviewer 1 Comments for the Author:

Major points to address

1. There is only a small difference in timing (~8%) between meiosis exit in mutants relative to controls or each other. Can the authors provide information to assure the reader that the synchronization has the precision to detect such a small timing difference? Are the strains sufficiently identical to remove concern about genetic background effects on the timing? Related, the inference of timing of CycB expression in Figure 1 G-I would be more convincing if the figure showed the cell-types where expression is detected (i.e. by morphology) or if the location/spacing of CycB expression was quantified.

The *lut* hs-Bam time-course flies differ from the control (vs. wt hs-Bam time-course flies) on chr 2 (location of *lut*), and the *rbp4* hs-Bam time-course flies differ from the control (vs. wt hs-Bam time-course flies) on chr. 3 (the location of *rbp4*). This means that the 8% difference in the timing of entry into the meiotic divisions in both *lut* and *rbp4* (vs. wt) is likely not due to a <u>shared</u> difference in genetic background between those two strains and the control strain in the hs-Bam time-course.

For Figure 1G-I, cell morphology is sacrificed to the IF conditions, especially the fixation step in methanol, so we cannot provide insight into germ cell stage in those particular samples. Instead we addressed the point by immunostaining for CycB protein in the heat-shock time-course to get granular detail on the timing of expression (Figure 3) - much more than what we would achieve by measuring the distance from CycB- expressing cysts to the tip of the testis or the edge of the spermatogonial zone.
Measuring the distance between CycB-positive cysts and the apical tip cannot be used because if a strain has elongating spermatids present, they push the early germ cell stages up against the tip, while in strains without robust spermatid elongation, the early germ cell stages spread away from the tip much more than in wild type.

2. The co-immunoprecipitations are done on extracts. It seems possible that the proteins interact in vivo within different complexes that are incorrectly amalgamated in Fig. 2c, the title of the paper, and elsewhere. Perhaps some Rbp4 interacts with the 3' UTR of CycB mRNA, and other Rbp4 is in a complex with Lut. Can the authors provide evidence that the proteins always co-localize in the cell with each other and with CycB mRNA?

We have removed all speculation that Lut and Fest are recruited to the *cycB* 3'UTR via Rbp4. This includes deleting the model images from Figures 2 and 8. In addition, since we cannot provide

© 2023. Published by The Company of Biologists under the terms of the Creative Commons Attribution License (https://creativecommons.org/licenses/by/4.0/).

data demonstrating that the proteins all co-migrate in a uniform peak in velocity sedimentation or sizing columns, we have removed “complex” from the title and throughout the manuscript.

3. The authors need to establish that Cyclin B expression is sufficient to drive the meiotic program – whether through citations or experiments.

In the original version, we described premature CycB expression as “permissive but not sufficient” for driving early cell cycle entry. We have re-written the discussion to make this clearer. We do not claim that expression of CycB is sufficient to drive entry into the meiotic divisions. Indeed our data from *rbp4* mutants indicate that CycB expression is clearly not sufficient in young and mid-stage spermatocytes. CycB expression is not sufficient in the classical literature on control of the mitotic cell cycle, where activation of Cdk1 by removal of inhibitory phosphorylation is also required.

Minor points to address

1. Aspects of the data/writing that could be better described/explained

a. The band in the negative control in Figure 2B seems problematic for their interpretation.

We have removed this panel (a reciprocal IP) from Figure 2, as reviewer #2 didn’t think it added much and wanted it removed (or moved to SOM).

b. Figure 3 A-D – there is an apparent expansion of CycB staining in spermatogonia at the later time points

We have added an explanation that this is the result of an expansion of the spermatogonial population itself due to the *bam* genetic background, once Bam protein is no longer being provided by heat shock (described in Materials and Methods).

c. Line 189-190 – more explanation of what this implies, full name, references

We added the full name and references for SYNCRIP (lines 207-8 of the revised manuscript)

d. Fig 4E-F “at least one cyst” is a reasonable way to quantify this data but please, at least qualitatively, describe how similarly the cysts behaved – they would be expected to be synchronized.

Under our heat-shock time-course conditions, the differentiating spermatocyte cysts are meta-synchronous, not totally synchronous; this is especially noticeable for short- duration events like the meiotic divisions. At 48h PHS, all spermatocytes are polar; at 72h PHS all spermatocytes have moved beyond the polar stage. However, at 108h PHS, some cysts might be in meiosis I, others may be in meiosis II, and yet others may contain early round spermatids. This intra-testis asynchrony was observed in all three genotypes scored for Figure 4. For this reason we chose to score the leading edge of the distribution of cysts entering and exiting meiotic division for Figure 4. We have added this info to the Materials & Methods.

e. More explanation/justification is needed for why expression of Syp in heads in the syp mutant is described as surprising.

We cut that sentence as the signal from one western blot was not sufficiently quantitative to make that conclusion.

2. Suggestions for tightening up the writing:

a. Refer to the timings in a consistent way. “PHS” is a great way to indicate that you are using and referring to the bam-HS system, but you only use this on occasion. I would be more consistent i.e. in Figure 1D,E, Figure 3, 6, etc.

© 2023. Published by The Company of Biologists under the terms of the Creative Commons Attribution License (https://creativecommons.org/licenses/by/4.0/).

Thank you. We now utilize “PHS” throughout the manuscript.

b. Line 141 – The topic sentence of this paragraph could better distinguish the experiments in this paragraph from those directly above. i.e. you are now using the hs- Bam system to narrow in on the timing of the interactions

We have rewritten the topic sentence to make the purpose of the experiment clearer.

c. Line 142 – Suggest mentioning/contrasting the CycB RNA expression pattern here.

We have added smFISH images showing *cycB* RNA for 72h and 104h PHS to Figure 2, underneath the corresponding anti-CycB panels.

d. The genes in the proposed seemed to arise out of the blue – please describe better why they were tested/studied f. The study of the genes in this paper seems to come out of left field. I would appreciate more explanation of how you arrived at these genes.

We have added content at the start of both the Lut and Syp sections to explain how these genes first caught our attention. Lut was identified as an interesting candidate from IP-MS analysis of proteins that interacted with Rbp4 and Fest. Syp was identified in an RNAi screen of predicted RNA-binding proteins whose transcripts were upregulated in spermatocytes.

e. Paragraph 189-201 vs 202-207 – Slightly different language is used to describe the same thing. If you mean that they are the same, you can write this more concisely. If you mean that they are different, please clarify how.

The first paragraph describes the expression from the four promoter options; the second paragraph, from the four C-terminal forms. We could collapse this into one paragraph but in doing so we think it risks making the complex locus confusing.

3. Figure 2F : “10% input” across the top left vs bottom right – I’m assuming the top left is a typo?

Thank you. We deleted the errant label.

4. The lut CRISPR mutant has not been demonstrated to be a null. You can refer to it as a “likely null” or give it an allele number.

We have changed “null” to “loss-of-function” in the results. We will refer to it as “ lut^1 ” when talking about the allele, and continue using “*lut*” as shorthand for lut^1/Df, as noted.

5. I don’t follow what the arrows are supposed to show in Fig 7A’-D’

We have removed a stray arrow from panel A’, changed the arrows in D to arrowheads and rotated them so that the reader’s eye is not tempted to correlate them with the arrowheads in D’, and updated the figure legend so that it says more precisely what arrowheads and arrows are pointing to (nucleoli, and the outside edge of relevant nuclei, respectively).

6. The sample shown in Figure 9K looks poorly fixed, which can lead to higher background staining. I’m concerned about the quantification of expression levels from samples such as this.

We have updated Figure 9 with new *cycB* smFISH images, plus smFISH against *loopin- 1* as an internal control. At the time of this experiment, we assumed *loopin-1* would be a negative control; Syp easyCLIP data obtained much later indicated that the *loopin-1* RNA is itself bound by Syp (Figure S3). That result made our finding that *loopin-1* RNA levels were reduced in the *syp* mutant (Figure 9) less surprising.

7. In Fig8A it looks like syp is upregulated in rbp4-/-. Could this indicate

© 2023. Published by The Company of Biologists under the terms of the Creative Commons Attribution License (https://creativecommons.org/licenses/by/4.0/).

competitive binding with Fest?

For this experiment we used more *rbp4* testes (77 pairs) than wt (55 pairs), as *rbp4* testes appear somewhat smaller than wt by eye. This may account in part for why there is more eYFP-Syp in the input and in the IP lanes. We therefore cannot conclude that Syp protein is upregulated in *rbp4* mutant testes.

******* Reviewer 2 Advance Summary and Potential Significance to Field:**

However, several points should be addressed prior to publication.

Reviewer 2 Comments for the Author:

The study has many moving parts with 10 figures and 2 supplemental figures. The transition from the Lut data (Figs 1-4) to the Syp data (Figs 5-9) should be improved. Simplifying and consolidating the figures may result in a clearer and more cohesive presentation of the overall story.

One of the primary weaknesses of the paper is that the authors do not provide strong evidence that these factors directly bind to the 3'UTR of cyclin B in vivo. Beyond the biotinylated pulldowns shown for Syp, the authors should perform RNA- immunoprecipitation for Rbp4, Fest, Lut, and Syp in the presence of crosslinking agents to show that these factors indeed interact with endogenous cyclin B mRNA.

We have added UV cross-linking immunoprecipitation (CLIP) data showing that Syp protein expressed in spermatocytes directly binds the *cycB* RNA (and others) *in vivo*. In a pilot CLIP experiment with each of the four proteins, Syp and Rbp4 showed robust RNA-binding (see Figure S3A for Syp), Lut showed faint RNA-binding, and Fest did not bind RNA. Because there were other RNA-positive bands above Rbp4-eYFP in the membrane for the pilot experiment, we did not expect to get a clean result from an Rbp4-eYFP easyCLIP library.

However, based on the promising preliminary result with Syp, we did easyCLIP for eYFP-Syp followed by sequencing the resulting cDNA libaries and found that Syp bound to both the 5' and 3'UTRs of the *cycB* RNA (Figure 5Q). Syp bound the 5' and/or 3'UTRs of a number of other RNAs as well. However, the binding appeared selective: compared to a set of spermatocyte-expressed genes with similar expression levels to *cycB*, *cycB* bound more Syp than most of the RNAs (see histogram in Figure S3C).

Additional structure/function analysis would bolster the claim that Rbp4, Fest, Lut and Syp form a complex in which Fest acts as a scaffold. Specific mutations in Fest that disrupt its interactions with other complex members should be made and tested for functional significance.

Absent co-sedimentation experiments or co-migration in sizing columns, we cannot prove that Rbp4, Fest, Lut, and Syp are in one complex. The small amounts of material from hand-dissected testis make such biochemical assays extremely difficult. In light of this, we have removed "complex" from the title and throughout the paper, also removing the ball-and-stick diagrams from the figures.

In terms of structure/function studies of Fest, we built transgenic flies carrying an HA- tagged version of Fest that contained only the conserved C-terminal half of the protein (Gly264-Y513), in hopes of testing whether this chunk of Fest was sufficient to bind one or more of Rbp4, Lut, and Syp. The protein appeared to be expressed normally by immunostaining but frustratingly did not show up <u>at all</u> in our IP-WB experiment, suggesting that it may have aggregated with insoluble material post-lysis.

We feel that more analysis (especially point mutations) is beyond the scope of this paper.
Does the HA-lut transgene first presented in Figure 1 rescue the lut mutant phenotype? If so, these data should be presented. Are lut males completely sterile? A brief description of the overall fertility of these mutants should be included in the paragraph describing the generation of the null mutant (lines 119-126).

© 2023. Published by The Company of Biologists under the terms of the Creative Commons Attribution License (https://creativecommons.org/licenses/by/4.0/).

We have added the information that HA-Lut does rescue the *lut*[1]/*Df* phenotype to the Materials and Methods.

In Figure 2A, treatment with RNAse appears to increase the interaction between Lut and Fest. Do the authors have an explanation for this?

We were also intrigued by this, but we do not have an explanation, unless it has something to do with the modest RNA-binding ability of Lut as observed in the CLIP pilot.

The IP presented in Figure 2B is relatively weak (IP shows lower levels of HA-Fest relative to 10% input). The authors could consider moving this panel into supplemental data or removing it altogether.

We have removed the panel from Figure 2.

Further biochemical experiments are needed to support the model presented in Fig 2C. It remains unclear whether all three proteins co-assemble into a single complex or whether sub-complexes form.

Figure 2A indicates that Rbp4 and Lut interact via Fest, suggesting that all three exist in a complex at some point. We agree that we have not shown that Lut and Fest are in a complex with the *cycB* RNA, so we have removed the model diagram from Figures 2 and 8 and have changed the text to be more conservative, removing mention of a “complex.”

The quality of the image presented in Fig 2E does not match other images throughout the paper. The authors should provide another image that convincingly shows Cyclin B expression at 104 hrs after hs-bam induction.

We have repeated the experiment and included the new images in Figure 2 (now B,C).

In Fig 2F- the “10% input” at the top of the panel may be misplaced.

Thank you. We have deleted the errant label.

For Figure 3- The authors should provide data describing the levels of cyclin B mRNA in the controls and mutants at all the timepoints. Some of the control data are presented in figure 6. Knowing the RNA levels in the mutants in Fig 3 would strengthen the interpretation of these data.

We have added these data; see Figure S1. (smFISH for wt, *lut*, and *rbp4* at the same timepoints in Fig 3)

The authors state, “In lut mutants, however, round spermatids typically had four nuclei and one large mitochondrial derivative per cell (Fig. 4H), suggesting complete failure of cytokinesis after meiosis I and II. In contrast, the rbp4 mutant showed evidence of much milder cytokinesis defects (Fig. 4I, arrows).” Given that cyclin B protein expression accumulates at earlier times in the rbp4 mutant relative to lut, can the authors provide a further explanation of how the lut phenotype relates to misexpression of Cyclin B protein?

We have added speculation to the Discussion that Lut may have other targets besides *cycB* that are not shared with Rbp4. Haynes et al (1997) indicated that “a few” (their words) proteins likely encoded by RNAs repressed by Rbp4 appeared in a 2D gel of *rbp4 boule* testis extract but were absent from *boule*. We suspect that Lut may have other roles or targets that are not shared by Rbp4.

In panels Fig 8B and Fig 8D, the levels of Syp appear to increase in the absence of rbp4 and fest respectively. Do the authors think each of these proteins influence the levels of one another?

© 2023. Published by The Company of Biologists under the terms of the Creative Commons Attribution License (https://creativecommons.org/licenses/by/4.0/).

As noted in our response to Reviewer #1, more pairs of *rbp4* testes (77 *rbp4* vs. 55 wt) were used in this experiment. This may have contributed to the excess of eYFP-Syp protein in 8A. Beyond that, we do not know whether Syp expression is negatively regulated by Rbp4. Because the mutant testes have different ratios of cell types compared to wild-type testes, one cannot draw conclusions about relative levels of expression of a protein in testes from one genotype compared to another from bulk tissue assays like western blots.

We opted to use more *fest* testes in Figure 8C to get the Rbp4-HA levels to roughly match each other for this experiment, at the expense of having equal levels of Syp (the same goes for Figure 8D and HA-Lut). Note that *fest* mutant germ cells are quite odd compared to *rbp4* and *lut*, suggesting that Fest likely has a role independent of Rbp4 and Lut that is related to microtubule growth/organization.

Does Lut influence the levels and/or interactions of Fest and Syp?

We performed co-immunoprecipitation (co-IP) assays for Fest and Syp in wt vs. *lut* mutant testis extracts and found that HA-Fest co-IPed with eYFP-Syp in anti-GFP immunoprecipitations at roughly similar levels in the two genetic backgrounds. However, because eYFP-Syp did not immunoprecipitate well in the *lut* background, the result is not quite publication-quality, and we would prefer to leave it out of the paper.

Data presented in figure 9 suggest that Rbp4, Lut, and Syp are needed for the stability of cyclin B mRNA. Why these levels appear to increase in the lut syp double mutants (Fig 9K) relative to the single mutants is unclear. Attempting to quantify mRNA levels using mean fluorescence is problematic without internal controls. Additional methods should be used to make this point because the presented images do not appear to match the quantification provided in Fig 9M. Statistics are needed in Fig 9M as well.

We have updated Figure 9 with a repeat of the smFISH that includes *loopin-1* as an internal control. Levels of *loopin-1* were lower in the *syp* mutant; this result was unexpected at the time but was not surprising after we discovered later by easyCLIP that the *loopin-1* RNA was a target of Syp binding (Figure S3).

The models presented in Figure 10 suggest that Lut represses cyclin B translation independent of rbp4. Do the authors mean to suggest that the physical interactions between rbp4 and lut (bridged by Fest) described in Fig 2 are not functionally significant? Further discussion along these lines is warranted.

As noted earlier, we have not been able to prove that Lut and Fest are complexed with Rbp4 (or with Syp, for that matter) on the *cycB* RNA, and we have re-worded the text conservatively in accordance with that.

<u>If</u> we had shown that Lut and Fest associate with the *cycB* RNA, we could speculate that Rbp4 plays two roles: a Lut-<u>independent</u> early role (54-94h PHS) and possibly a Lut-<u>aiding</u> late role (94h-102h PHS), in which Rbp4 contributes to the full recruitment of Lut (via Fest) to the *cycB* RNA. Our previous ball-and-stick model (now removed from the manuscript) had Fest associating with the *cycB* 3'UTR via both Rbp4 and Syp; in our experience, the Rbp4-Fest interaction is stronger than Syp-Fest, so Fest (and Lut) might in theory not be recruited as efficiently to the *cycB* 3'UTR in an *rbp4* mutant as in wt.

Again, we have not been able to show this, so this speculation is for reviewers' eyes only.

<u>Second decision letter</u>

MS ID#: DEVELOP/2023/201709

© 2023. Published by The Company of Biologists under the terms of the Creative Commons Attribution License (https://creativecommons.org/licenses/by/4.0/).

MS TITLE: Cell-type-specific interacting proteins collaborate to regulate the timing of Cyclin B protein expression in male meiotic prophase

AUTHORS: Catherine C Baker, Lorenzo Gallicchio, Neuza R Matias, Douglas F Porter, Lucineh Parsanian, Emily Taing, Cheuk H Tam, and Margaret T Fuller
ARTICLE TYPE: Research Article

I am happy to tell you that your manuscript has been accepted for publication in Development, pending our standard ethics checks.

Reviewer 1

Advance summary and potential significance to field

The authors show that Drosophila spermatogenesis protein Lutin and spermatogenesis isoforms of the Syp protein regulate the timing of meiosis exit and translational expression of Cyclin B. They also show that Lutin and Syp interact through the agency of another protein, Fest. RNA is not required for this interaction. This work expands knowledge of the control of cyclin B translation and of the contributors to the timing of meiotic exit in Drosophila.

Comments for the author

The authors' revisions addressed my concerns.

Reviewer 2

Advance summary and potential significance to field

This manuscript describes the characterization of several RNA binding proteins that physically interact to control the translation of cyclin B within the developing germline of Drosophila males. Cyclin B is translationally repressed in immature Drosophila spermatocytes and previous work from the lab showed that Rbp4 and its partner Fest can bind to elements within the 3'UTR of cyclin B mRNA. The work presented here extends these earlier findings and shows that another spermatocyte-specific protein, Lut also participates in the repression of cyclin B during an 8 hour window of time, just before spermatocytes are mature. In addition, spermatocyte-specific isoforms of Syncrip (Syp) regulate the normal expression of cyclin B and entry into meiosis. The authors provide evidence that Lut and Syp interact with Fest in an RNA-independent manner. New data show that Syp protein directly interacts with sequences in the 5' and 3' UTR of cyclin B mRNA. Together, these results provide new insights into a regulatory mechanism that controls the timing of male meoitic prophase.

Comments for the author

The authors have addressed all of my previous concerns. I find the manuscript acceptable for publication.

© 2023. Published by The Company of Biologists under the terms of the Creative Commons Attribution License (https://creativecommons.org/licenses/by/4.0/).
